# Stabilized and Controlled Release of Radicals within Copper Formate-Based Nanozymes for Biosensing

**DOI:** 10.1021/acsami.3c08326

**Published:** 2023-09-07

**Authors:** Yue Zhou, Xiaohua Chen, Shaoqi Zhan, Qiang Wang, Feng Deng, Qingzhi Wu, Jian Peng

**Affiliations:** † State Key Laboratory of Advanced Technology for Materials Synthesis and Processing, and School of Chemistry, Chemical Engineering and Life Science, Wuhan University of Technology, Wuhan 430070, China; ‡ Department of Laboratory Medicine, Nanfang Hospital, Southern Medical University, Guangzhou 51015, China; § Department of Chemistry—BMC, Uppsala University, BMC Box 576, Uppsala S-751 23, Sweden; ∥ State Key Laboratory of Magnetic Resonance and Atomic and Molecular Physics, Innovation Academy for Precision Measurement Science and Technology, Chinese Academy of Sciences, Wuhan 430071, China

**Keywords:** Cuf-TMB nanozymes, ·OH radicals, peroxidase-like activity, radical stability, biosensors

## Abstract

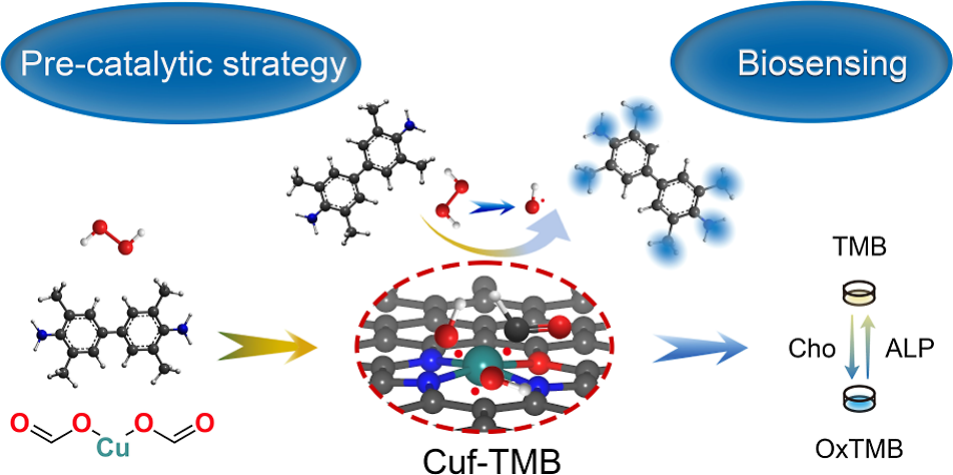

Fenton-like radical processes are widely utilized to explain catalytic mechanisms of peroxidase-like nanozymes, which exhibit remarkable catalytic activity, cost-effectiveness, and stability. However, there is still a need for a comprehensive understanding of the formation, stabilization, and transformation of such radicals. Herein, a copper formate-based nanozyme (Cuf-TMB) was fabricated *via* a pre-catalytic strategy under ambient conditions. The as-prepared nanozyme shows comparable catalytic activity (*K*_m_, 1.02 × 10^–5^ mM^–1^; *K*_cat_, 3.09 × 10^–2^ s^–1^) and kinetics to those of natural peroxidase toward H_2_O_2_ decomposition. This is attributed to the feasible oxidation by *OH species *via* the *O intermediate, as indicated by density functional theory calculations. The key ·OH radicals were detected to be stable for over 52 days and can be released in a controlled manner during the catalytic process *via in situ* electron spin-resonance spectroscopy measurements. Based on the understanding, an ultrasensitive biosensing platform was constructed for the sensitive monitoring of biochemical indicators in clinic settings.

## Introduction

Nanozymes, a class of nanomaterials exhibiting intrinsic enzyme-like properties, have drawn increasing attention due to their enhanced catalytic activity, cost-effectiveness, and stability. These merits enable their diverse applications, ranging from biological analysis to disease diagnosis and the development of biomedicine.^1−9^ Typical nanozymes capable of imitating the catalytic activity of peroxidase (POD) have been discovered since Yan’s group unexpectedly showed the intrinsic POD-like activity of magnetic Fe_3_O_4_ nanoparticles.^10^ To date, POD nanozymes can be categorized into metal-based nanozymes (Pd,^11^ Au,^12^ Ag,^13^*etc.*), metal-oxide or sulfide-based nanozymes (Fe_3_O_4_,^14^ CeO_2_,^15^ MoS_2_,^16^*etc.*), carbon-based nanozymes (graphene oxide,^17^ g-C_3_N_4_ nanosheet,^18^*etc.*), single-atom (FeN_3_P single-atom nanozymes^19^), or metal–organic complex nanozymes (copper/carbon hybrid nanozyme).^20^ Metal–organic complex nanozymes, among other types, possess well-defined structures that allow them to imitate the highly evolved catalytic center of natural enzymes at the atomic level and serve as favorable alternatives to traditional enzymes.^21−23^ Despite their significant development, nanozymes are frequently seen as fascinating and enigmatic “black boxes” due to the limited understanding of their catalytic reaction pathways.^24−26^ The Fenton-like mechanism, which involves the reaction of Fe^2+^ with hydrogen peroxide (H_2_O_2_) to generate hydroxyl radicals (·OH), is often applied to describe the catalytic mechanism of nanozymes.^27−29^ In this reaction pathway, nanozymes typically attach to and react with the initial substrate H_2_O_2_ to produce intermediate ·OH radicals, which will subsequently oxidize hydrogen donors such as 3,3′,5,5′-tetramethylbenzidine (TMB).^30,31^

During the reaction process, the ·OH radicals produced from H_2_O_2_ are considered one of the possible factors for the high catalytic activity of nanozymes.^32^ Gao and other studies have proved that the catalytic effect of Fe_3_O_4_ nanozyme arises from the generation of the ·OH,^33^ indicating the key substance of the generated ·OH for exerting POD-like activity. The produced ·OH radicals can be stabilized on the nanozyme^34,35^ and have been monitored by electron spin-resonance spectroscopy (ESR) in the Fenton-like reaction. For instance, the ·OH radicals in the CeO_2_ nanozyme system were monitored to be stable for 30 min.^36^ The stabilized free radicals could subsequently oxidize the substrates, for instance, TMB. More importantly, the stronger the·OH radical intensity, the better the nanozyme catalytic performance.^33^ Despite these studies on the catalytic behavior and kinetics of nanozymes, the accurate mechanism instead of mimicking Fenton-like reaction pathways underlying the POD-like activity of nanozymes is still undisclosed.^37^ Moreover, the lack of clarity in the formation and transformation processes of the ·OH or other radicals and their influence on the catalytic activity and kinetics has greatly limited researchers to design new structures of nanozymes with enhanced catalytic activity.

Herein, we developed a pre-catalytic strategy to engineer copper formate (Cuf)-amine-based nanozymes (Cuf-TMB NPs), in which a substrate was introduced to the system during the preparation of the nanozyme, to address the challenge. The Cuf-TMB nanozyme showed equivalent POD-like and kinetics to natural POD enzymes under ambient conditions. Their outstanding POD-like activity was explained *via* a proposed ·OH stabilization, transformation, and controlled release route, validated by manipulating the electronic and geometric structures of the Cuf active center with different amine ligands. The key intermediate of ·OH was monitored to be stable for up to 52 days. Additionally, an ultrasensitive biosensing platform using the developed Cuf-TMB nanozyme was constructed for cholesterol and alkaline phosphatase (ALP) detection in serum (Scheme S1).

## Materials and Methods

### Reagents

Cuf, cholesterol oxidase (Chox), cholinesterase (Chex), l-ascorbic acid phosphate trisodium (AA2P), copper pyrophosphate, lysine, threonine, tryptophan, dopamine hydrochloride, aniline, 3,5-dimethylpiperidine, 3,5-dimethylaniline, 2,3-dimethylaniline, 4,4-dimethylaniline, 3,4-dimethylaniline, 2-trimethylaniline, 2,5-dimethylaniline, *o*-phenylenediamine (OPD), horseradish peroxidase (HRP), and 2,4-dimethylaniline were purchased from Macklin Biochemical Co., Ltd (Shanghai). Glucose oxidase (GOx) and ALP were purchased from Sigma-Aldrich. Glucose, lactose, fructose, maltose, sodium acetate, glacial acetic acid, copper sulfate, l-valine, and l-ascorbic acid were purchased from Sinopharm Chemical Reagent. TMB, copper perchlorate hydrate, and cholesterol were obtained from J&K Scientific. Tris–HCl buffers at pH 6.8 and pH 7.6 were obtained from Biosharp. Copper tartrate, hexadecylamine, dodecylamine, copper acetate, copper oxalate, copper citrate, l-cysteine (Cys), glycine, threonine, histidine, and lysine were purchased from Aladdin. Di-azo-aminobenzene (DAB) was obtained from Beyotime Biotechnology Co., Ltd. Fe_3_O_4_ nanoparticles, C nanoparticles, and CuO nanoparticles were purchased from XFNANO. Cu_2_O was obtained from Beijing Zhongke Keyou Technology Co., Ltd. 5,5-Dimethyl-1-pyrroline-*n*-oxide (DMPO) was purchased from Dojindo Molecular Technology Co., Ltd. All the chemicals were used as received without further purification. The ultrapure water used in all experiments was made by passing through an ultrapure purification system. Human serum samples were collected from triglyceride patients at the general hospital of the central theater command. The study was ethically approved under the number [2022]034-01 on March 1, 2022.

### Synthesis of Cuf-TMB NPs

The synthesis process of Cuf-TMB NPs could be divided into three steps: first, 0.4 g of sodium acetate was dissolved in 300 mL of DI water, and the pH of the solution was modulated to neutral using acetic acid to produce a buffer solution. Second, 100 mL of H_2_O_2_ (100 mM) was added into the buffer solution, followed by the addition of 100 mL of the Cuf (5 mM) solution under stirring. Third, 2 mM of TMB dissolved in 100 mL of ethanol was added into the above mixture under stirring at room temperature. The reaction lasted for 30 min before the Cuf-TMB NP suspension was formed. Finally, the Cuf-TMB NP suspension was dialyzed overnight to remove excess H_2_O_2_, Cuf, and other byproducts before the following catalytic measurements. After the reaction, the Cuf-TMB NP supernatant was dialyzed against 3 L of water and 0.6 L of ethanol for 12 h to remove free ions and small molecules, and then the purification products were centrifugated and washed three times. Purified Cuf-TMB was obtained by freeze-drying.

### Loading of Cuf-TMB NPs on Agarose Hydrogel

1 g of agarose was dissolved in 100 mL of water and heated to boiling until the solution became fully transparent. After cooling the agarose solution to 60 °C, it was mixed with the Cuf-TMB NPs (suspension in different volume ratios and mixed uniformly before being put into 6-well plates). The volume ratios of Cuf-TMB NPs to agarose were 1:9, 2:8, 4:6, 6:4, 8:2, and 9:1, corresponding to no. 1–6 samples, respectively. After cooling the Cuf-TMB NPs/agarose mixture to room temperature, the Cuf-TMB NP hydrogel was produced.

### Characterization

The crystal structure of the Cuf-TMB NP samples was examined by X-ray diffraction (XRD, Rigaku Smart Lab). The morphological characterization of the prepared samples was obtained by scanning electron microscopy (SEM, HITACHI SU8010), transmission electron microscopy (TEM, JEM-1400), and high-resolution TEM (HRTEM, Thermo Scientific Talos F200X G2). The molecular structure and bond information of the Cuf-TMB NPs were obtained with Fourier transform infrared spectroscopy (FT-IR, Nicolet 6700) and laser confocal micro-Raman spectroscopy (Xplora PLUS). The composition and chemical state of the elements were characterized using X-ray photoelectron spectroscopy (XPS, Thermo Fisher Scientific K-Alpha). The concentration of metal content was determined by inductively coupled plasma atomic emission spectroscopy (Agilent 720ES). The electrochemistry behavior of the Cuf-TMB NPs was investigated by a three-electrode system with an electrochemical workstation (CHI-760E). The Cu K-edge X-ray absorption fine structure spectrum was collected at the Beijing Synchrotron Irradiation Facility (BSRF, 1W1B) using transmission mode. Athena and Artemis software packages were employed to process and fit the XAS data, respectively. The POD activity of Cuf-TMB NPs was tested by a UV-1900i Visible-near-infrared spectrophotometer (Shimadzu, Japan) and Microplate Reader (Thermo Multiskan GO 1510).

### POD-like Activity of Cuf-TMB NPs

1.5 mL of NaAc-HAc buffer solution (pH = 7), 500 μL of TMB solution (2 mM), 500 μL of H_2_O_2_ (10 mM), and 500 μL of Cuf-TMB NP suspension (0.068 mg/mL) were well mixed and kept at 20 °C for 5 min under shaking. The absorbance at 652 nm of the resulting solution was measured by using a UV-1900i Visible-near-infrared spectrophotometer or microplate reader. For the detection of H_2_O_2_, the concentration of TMB was fixed at 2 mM, and various concentrations (0–10 mM) of H_2_O_2_ solution were added to the reaction mixture. The absorbance at 652 nm was then recorded over time. Color reactions were recorded in time-scan mode by measuring the absorbance at 652 nm using a UV–visible spectrometer (UV-1900i).

### Density Functional Theory Calculations

Density functional theory (DFT) calculations were carried out using the Vienna ab initio simulation package.^38,39^ The electron–core interactions were described using the projected augmented wave^40^ method, and electron exchange–correlation was expressed at the general gradient approximation level with the Perdew–Burke–Ernzerhof functional.^41^ The plane-wave basis set with a cutoff energy of 500 eV was adopted. Spin polarization was used in all calculations. For structural optimization, a convergence threshold of 0.03 eV Å^–1^ was set in force, and the total energy converged to within 10^–5^ eV. Grimme’s method (DFT-D3)^42^ was utilized to account for van der Waals interaction during surface adsorption. The Gamma-point was considered for sampling the Brillouin zone during the calculations. The CuN_3_O and CuN_2_O_2_ models (total 97 atoms) were constructed with a layer of 7 × 7 graphene supercell to enclose a Cu atom. Carbon atoms directly coordinated with the Cu atom were replaced by nitrogen and oxygen atoms. The models included a vacuum region of 15 Å in the *z*-direction. A 2 × 2 × 2 *k*-point mesh with horizontal shifts was used for integration over the reciprocal space. The quasi-Newton^43−45^ method was used to obtain stable and transition-state structures. The VTST tool with the nudged elastic band^46,47^ method and an approximate convergence of 0.05 eV A^–1^ was used to locate the transition state. VESTA was used to visualize the molecular structure.^48^

### Identification of Radicals

The ESR was performed by a JEOL JES-FA 200 spectrometer at room temperature. The frequency is set at 9.1 GHZ, the modulation amplitude is 0.1 mT, and the microwave power is 5 mW. The raw materials were also characterized by ESR. For the capture of radicals, DMPO was used as a trapping agent. Cuf-TMB NP suspension was mixed with DMPO solution (1%), and the reaction mixture was measured by ESR. During the synthesis process of Cuf-TMB NPs, the DPMO was added, and the ESR spectra were collected *in situ* spanning from 6 to 66 min. The intensity of ·OH and ·CHO radicals was analyzed and compared. Samples were taken from Cuf-TMB NP reaction stock suspension at 1, 8, 18, 52, and 73 days during the reaction at ambient conditions before ESR spectra were collected. The stability of ·OH and ·CHO radicals was also evaluated by monitoring the ESR signals.

### Cholesterol Sensing

Cholesterol detection was carried out as follows: (1) 100 μL of cholesterol oxidase solution (5 mg mL^–1^) and 50 μL of cholesterol stock solution (dissolved in 5% Triton X-100) with different concentrations were added in a tube, which was kept at 37 °C for 30 min under shaking. (2) 200 μL of Cuf-TMB NP hydrogel, 50 μL of the incubated solution, and 50 μL of TMB solution (2 mM) were added into each well of a 96-well plate. (3) The absorbance was detected as described above.

Detection of human serum was carried out as follows:^49^ (1) sodium cholate (3 mM), 4-amino antipyrine (1.5 mM), cholesterol esterase (200 U L^–1^), cholesterol oxidase (100 U L^–1^), peroxidase (1.5 × 10^–4^ mM), and phenol (30 mM) were prepared in a phosphate buffer solution with pH 6.8. (2) 300 μL of enzyme reagent and 30 μL of human serum were mixed uniformly and kept at 37 °C for 30 min. (3) The absorbance of quinonimine at 500 nm was recorded by a spectrophotometer.

### Ascorbic Acid Detection

The determination of ascorbic acid (AA) was operated as follows: (1) 200 μL of Cuf-TMB NP hydrogel, 50 μL of AA solution with different concentrations, 50 μL of H_2_O_2_ (10 mM), and 50 μL of TMB solution (2 mM) were added into the 96-well plate. (2) The absorbance was detected as described above.

### Detection of Alkaline Phosphatase

The determination of ALP was operated as follows: (1) 100 μL of sodium AA2P (100 mM), 100 μL of different concentrations of ALP, and 50 μL of Tris–HCl (pH 8.0, 50 mM) were mixed and incubated at 37 °C for 40 min under shaking. (2) 200 μL of Cuf-TMB NP hydrogel, 50 μL of an incubated solution, 50 μL of H_2_O_2_ (10 mM), and 50 μL of TMB (2 mM) were added into a 96-well plate in turn. (3) The absorbance was detected as described above. Detection of human serum was carried out as follows:^50^ (1) reagent 1 consists of Tris–HCl buffer solution, 2-amino-2-methyl-1-propane, and magnesium chloride. Reagent 2 consists of a Tris–HCl buffer solution and *P*-Nitrophenyl phosphate. (2) 250 μL of reagent 1 and 5 μL of human serum were mixed uniformly at 37 °C. (2) 300 s later, 50 μL of reagent 2 was added to the mixed solution. (3) The absorbance of 405 nm was recorded over time using a microplate reader. ALP activity can be calculated by measuring the absorbance ascent rate of *p*-nitrophenol at 405 nm.

## Results and Discussion

### Synthesis and Characterization of Cuf-TMB NPs

**Figure 1:**
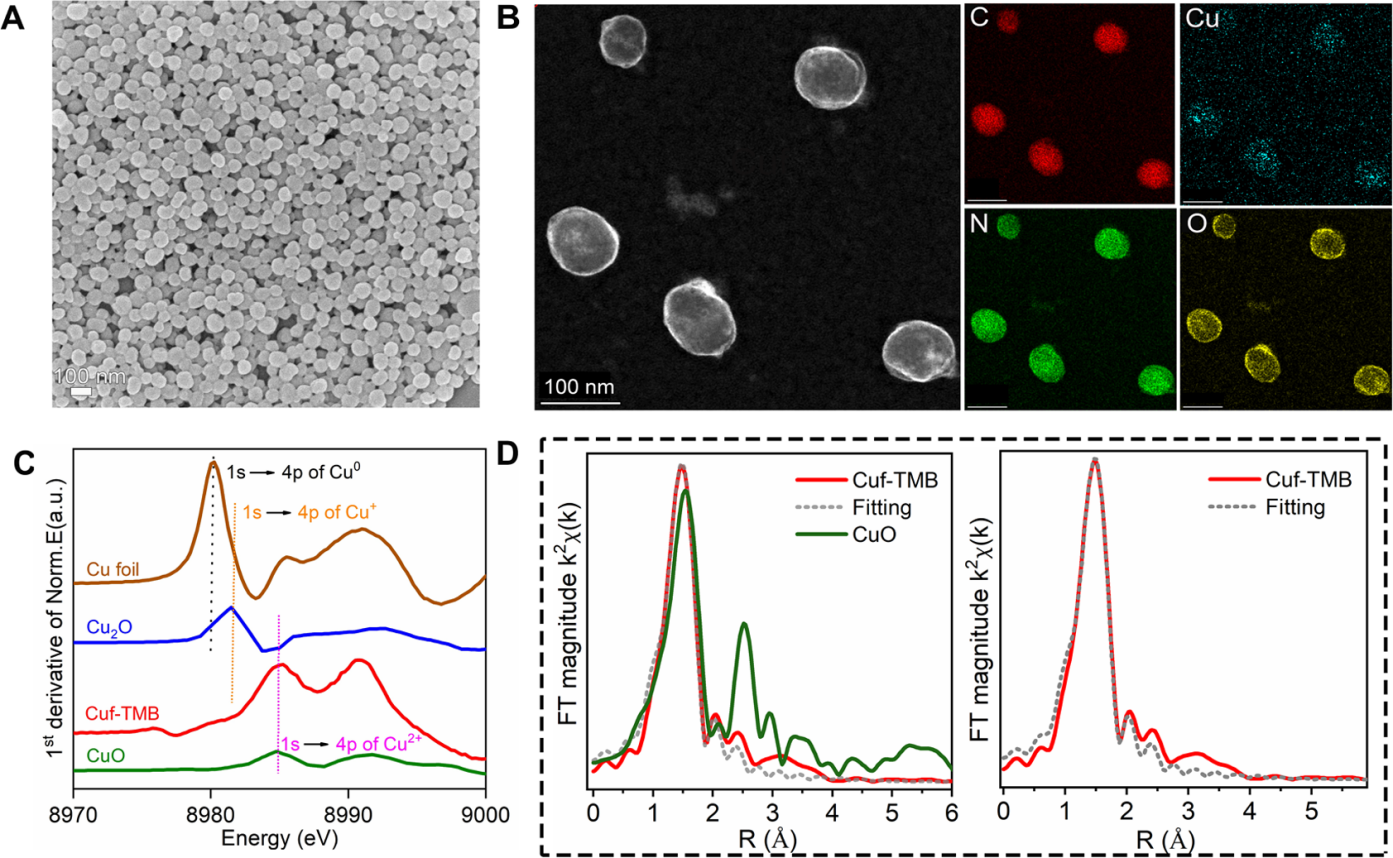
Characterization of Cuf-TMB NPs. (A) SEM image of Cuf-TMB NPs (scale bar, 100 nm). (B) High-angle annular dark field scanning transmission electron microscopy image and the corresponding energy dispersive spectrometer mapping images of Cuf-TMB NPs (scale bar, 100 nm). (C) XANES spectra of Cu foil, Cu_2_O, CuO, and Cuf-TMB NPs, with reference to the copper L-edge. (D) FT magnitude of the Cu K-edge EXAFS signal of Cuf-TMB NPs and CuO (left), and Cuf-TMB NPs before and after fitting (right).

Cuf-TMB NPs were prepared *via* a typical room-temperature stirring method (Scheme S1a). During the synthesis, *in situ* monitoring of pH and temperature showed a noticeable increase in temperature accompanied by a decrease in pH values upon the addition of TMB, indicating the synthesis was driven by the coordination of TMB to Cuf (Figure S1). Successful conjugation of Cuf and TMB was confirmed by Raman and FT-IR spectra (Figures S7, S8, Tables S1, S2), forming amorphous and spherical nanostructures with an average diameter of ∼80 nm (Figures 1A,B, S2–S6). XPS (Figure S9) revealed that Cuf-TMB NPs consist of Cu(II) coordinated by formate and TMB. The +2 valence state of Cu species was further proved by chemical shifts and edge slope from X-ray absorption near-edge structure (XANES) and FT extended X-ray absorption fine structure (EXAFS) (Figure 1C).^51^ From the EXAFS signal, the main peaks of the Cu atoms in Cu-N_*x*_ are located at ∼1.48 Å, corresponding to the Cu–N bond (Figure 1D). Elemental analysis showed a Cu content of 8.59% and a Cu/N molar ratio of ∼3, indicating that Cu(II) is coordinated by three TMB ligands and one formate (Table S3). The *in situ* cyclic voltammetry measurements of Cuf-TMB suspensions also confirmed the chemical environment of Cu(II) species and the existence of ·OH radicals in the suspension (Figure S10, Table S4).

### POD-like Activity of Cuf-TMB NPs

The synthesized Cuf-TMB NPs exhibit activity in oxidizing TMB and other POD substrates, such as DAB and OPD, with distinct color changes (Figure S11). Control experiments recorded using an ultraviolet–visible spectroscopy (UV–vis) spectrometer showed that only a combination of Cuf-TMB and H_2_O_2_ has obvious absorption at ∼652 nm, corresponding to ·OH radicals. This observation indicates the necessity of both Cuf-TMB and H_2_O_2_ for the POD-like activity of Cuf-TMB NPs (Figure S12). Atomic absorption spectroscopy of Cuf-TMB supernatant revealed an extremely low concentration of copper only 0.0039 mg L^–1^, compared to the initial amount of injected copper (78 mg L^–1^). The low concentration indicates that the impact of the supernatant copper is negligible. The POD-like activity of Cuf-TMB NPs was optimized under the conditions of ∼ pH 7, 20 °C, and 5 mM of Cuf concentrations (Figure S13). Under these optimal reaction conditions, Cuf, which will be suitable for biosensing applications under physiological conditions, was replaced by other Cu(II) salts to investigate the effect of different copper-based precursors on the POD-like activity. It was demonstrated that Cuf-TMB NPs showed much higher POD-like activity than other Cu(II) salt-derived NPs. The difference in activity is likely due to the smallest particle size distribution of Cuf-TMB NPs (Figures S14, S15).

**Figure 2:**
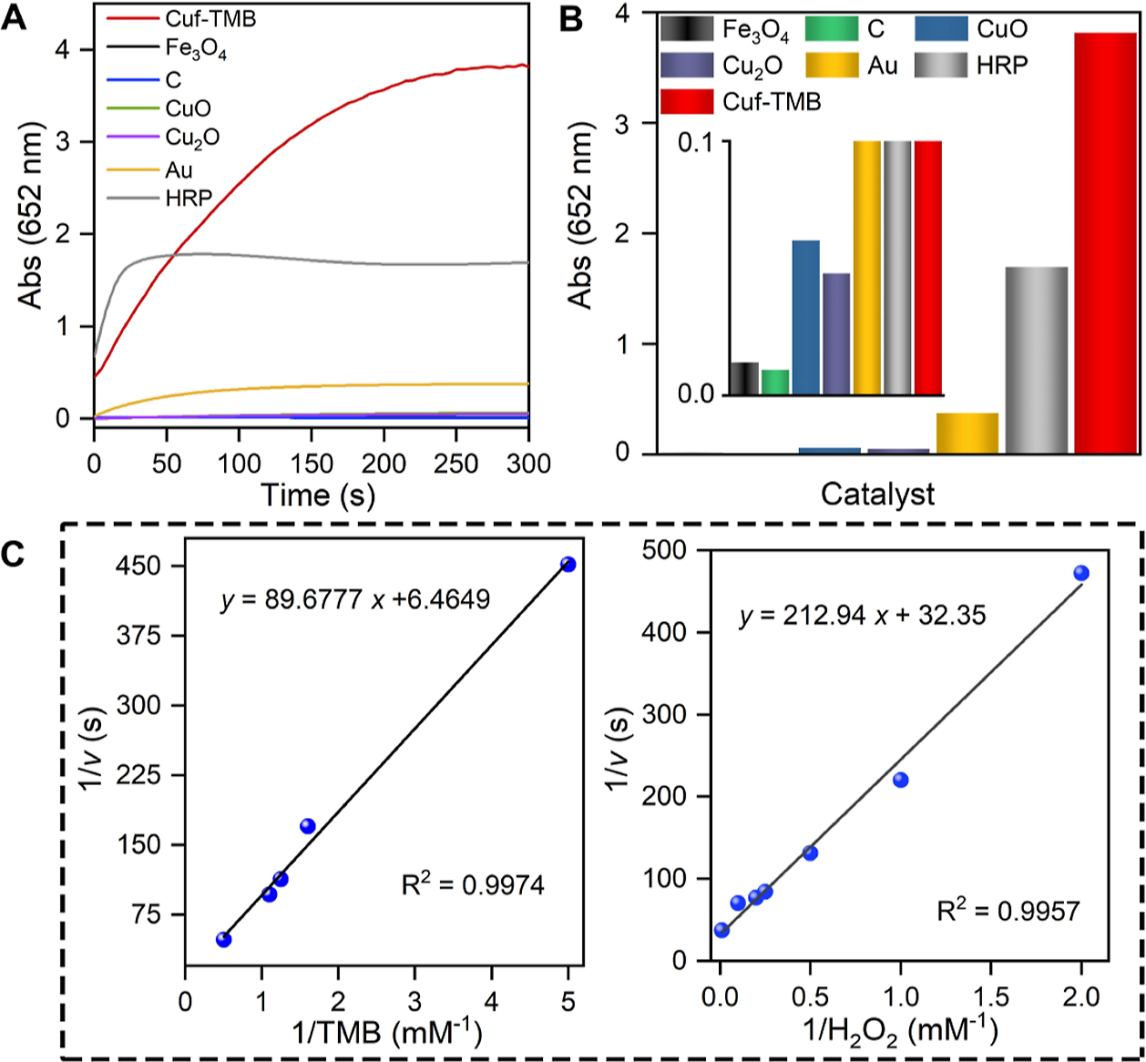
POD-like activity and kinetics of Cuf-TMB NPs. (A) Reaction-time curves of TMB colorimetric reactions for Cuf-TMB NPs, CuO NPs, Cu_2_O NPs, Au, C, Fe_3_O_4_ nanozymes, and HRP at pH 7. (B) Comparison of the specific activities of Cuf-TMB, CuO, Cu_2_O, Au, C, Fe_3_O_4_ nanozymes, and HRP at pH 7. Inset: The magnified absorbance at 652 nm of the nanozymes. (C) Double-reciprocal plots of activity of Cuf-TMB NPs at a fixed concentration of one substrate *versus* a varying concentration of the second substrate for TMB (left) and H_2_O_2_ (right).

The catalytic performance of Cuf-TMB NPs was further evaluated by comparison with other traditional nanozymes such as Fe_3_O_4_, C, CuO, Cu_2_O, and Au (Figure S16). The evaluation was conducted at pH 7 and 20 °C, with the concentration of the central metal atom as the basis (79 μg mL^–1^). The results showed that the Cuf-TMB NPs exhibited the best catalytic performance, which was 245, 318.5, 52.21, 66.35, and 8.47 times that of Fe_3_O_4_, C, CuO, Cu_2_O, and Au, respectively. The absorbance at 652 nm of the ·OH radical intermediate produced by Cuf-TMB, Fe_3_O_4_, C, CuO, Cu_2_O, and Au was 3.815, 0.013, 0.01, 0.0061, 0.048, and 0.376, respectively (Figure 2A,B). To further assess the catalytic oxidation of TMB, kinetic studies of Cuf-TMB NPs were conducted by varying the concentration of one substrate while maintaining the other at a saturated concentration. The obtained data were fitted by the Michaelis–Menten equation, obtaining a typical double-inverse Lineweaver–Burk plot. The plot revealed that Cuf-TMB NPs exhibited a good binding affinity for H_2_O_2_ and TMB, confirming their outstanding catalytic properties (Figure 2C). It should be noted that as-prepared Cuf-TMB NPs showed a high turnover number (TON) of 8.91 and turnover frequency (TOF) of 1069.72 h^–1^, comparable to that of the natural HRP enzyme (Figure S17). In comparison to traditional nanozymes, Cuf-TMB NPs showed a lower *K*_m_ (1.02 × 10^–5^ mM) (Tables S5–S7) but higher TON and TOF (Table S8), highlighting their superb catalytic efficiency.

### Mechanism of Catalytic Stabilization of Cuf-TMB NPs

**Figure 3:**
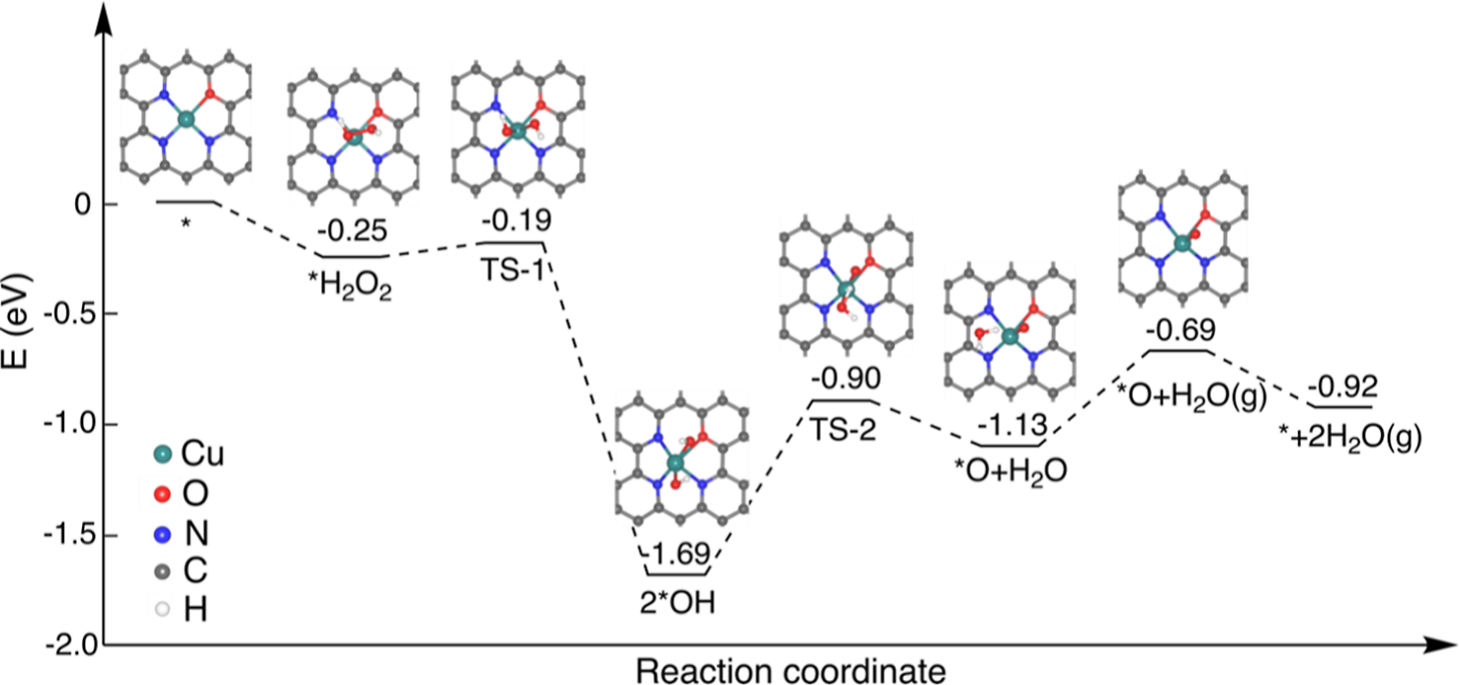
Energy panel of the reaction pathway on the CuN_3_O model. The energy graphic displays the most favorable chemical pathway of H_2_O_2_ dissociation into surface *O species that occurs under neutral circumstances.

The reasons behind the high POD-like activity of Cuf-TMB NPs were illuminated by DFT calculations. Calculations primarily focused on the formation of ·OH or ·O species from H_2_O_2_ reduction and the oxidation of POD substrates by the Cuf-TMB NPs, as the presence of ·OH radicals affects the activity observed from experiments. According to experimental characterizations, a structural model (CuN_3_O) of Cu coordinated with three nitrogen and one oxygen atom was constructed and optimized (Figure S18). As shown in Figure 3, the adsorption of H_2_O_2_ on the CuN_3_O is favorable with an adsorption energy of −0.25 eV. The following dissociation of the *H_2_O_2_ into two *OH has a downhill energy of −1.44 eV and a low barrier of 0.06 eV, indicating the stable *OH species is highly feasible to form. Subsequently, the H transfer between the two *OH species leads to the formation of an *O species and an *H_2_O molecule with a barrier of 0.79 eV. The desorption of the produced H_2_O molecule is endothermic by 0.44 eV, and the oxidation of POD substrates by surface *O species requires −0.23 eV [TMB + *O → oxTMB + H_2_O (g)] (Figure S19). Therefore, the overall uphill energy for the oxidation by the *OH species *via* the *O intermediate is 0.77 eV. This oxidation level is significantly lower than the direct oxidation by surface *OH species [TMB + 2*OH → oxTMB + 2H_2_O (g)], which necessitates 1.30 eV. Thereby, the formation of *O intermediate species from *OH species emerges as the key, lowering the energy barrier of the oxidation by *OH and leading to the high performance of POD-like activity exhibited by Cuf-TMB NPs.

**Figure 4:**
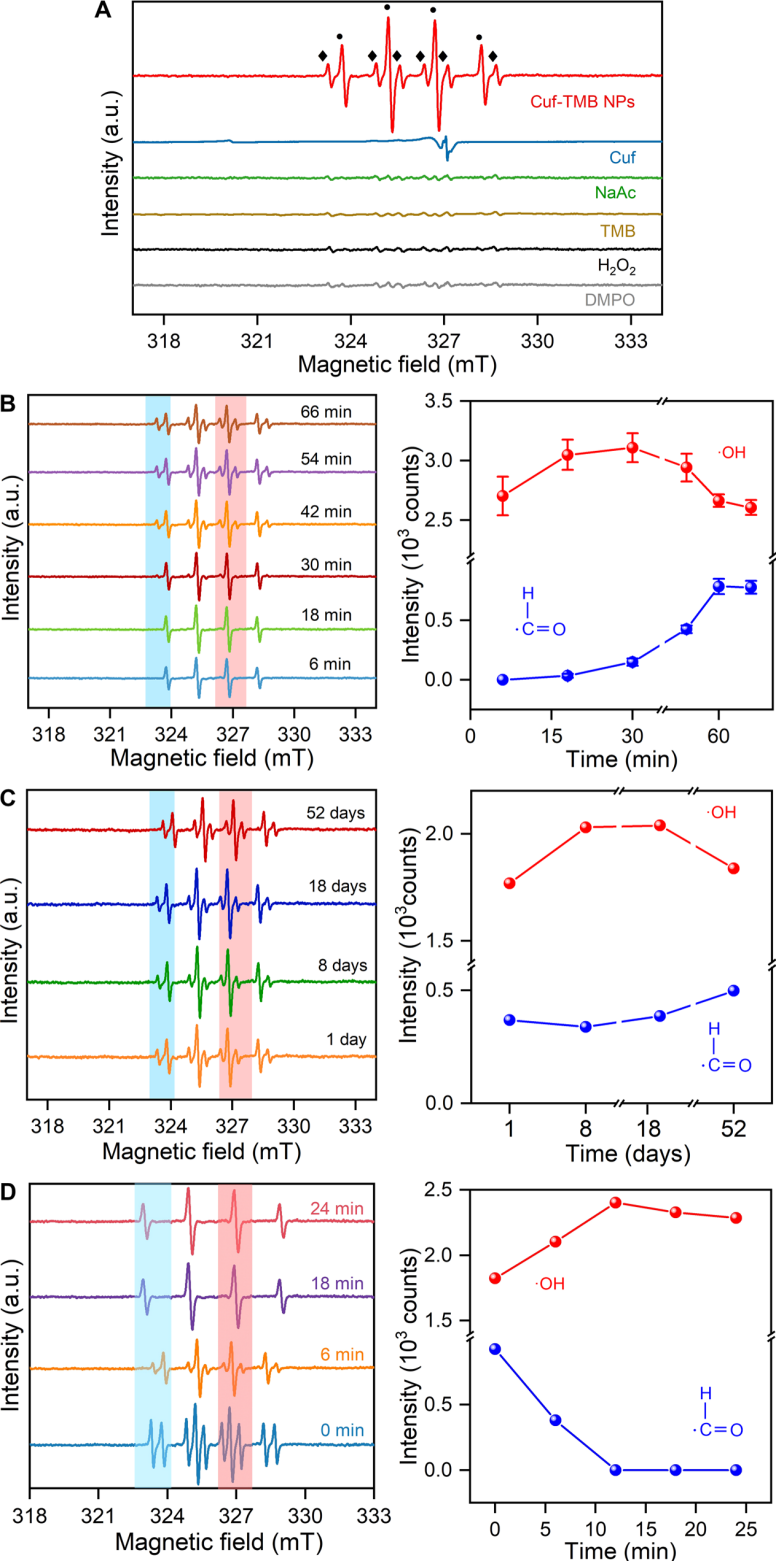
Formation and transformation processes of radicals. (A) ESR spectra of Cuf-TMB NPs and other controls through DMPO trapping. (B) *In situ* ESR probing of the synthesis process of Cuf-TMB NPs from 6–66 min. The intensity change of ·CHO and ·OH species was derived based on three independent measurements. (An average and a standard deviation of all intensity was reported) (C) ESR spectra of Cuf-TMB NP suspension during different aging times under ambient conditions. ESR intensity change of ·CHO and ·OH species. (D) *In situ* ESR spectra of Cuf-TMB NP suspension upon the addition of H_2_O_2_, indicating the transformation of ·CHO into ·OH upon H_2_O_2_ addition. The intensity changes of the ESR signal for Cuf-TMB NP suspensions upon the addition of H_2_O_2_ (10 mM) using DMPO trapping are shown (Red: The intensity of ·OH; Blue: The intensity of ·CHO). The increased intensity of ·OH with decreased intensity of ·CHO indicates the transformation of ·CHO into ·OH upon H_2_O_2_ addition.

To characterize key intermediates of radicals formed during the reaction, we recorded the ESR spectra of raw materials and products after adding DMPO, a spin-trapping agent of radicals, into the initial reaction mixture (Figures 4A and S20). The ESR spectra of H_2_O_2_ and TMB alone did not present obvious signals upon DMPO trapping. While in the Cuf-TMB NPs, distinct ESR signals in a 1:2:2:1 quartet pattern with a splitting of 1.5 mT were observed. These ESR signals were attributed not only to ·OH but also to ·CHO species,^52^ raising the need to clarify the generation of ·CHO species as the Fenton-like reaction typically produces ·OH. Therefore, ESR spectra were *in situ* collected during the reaction process of the mixture of TMB, H_2_O_2_, and Cuf until the equilibrium was reached. Figure 4B demonstrates that the signal corresponding to ·OH (a 1:2:2:1 quartet pattern) rapidly increased and reached a stable level within the first 30 min of the reaction. However, it gradually decreased thereafter. In contrast, the signal attributed to ·CHO (a 1:1:1:1:1:1 sextet pattern) appeared at 18 min and continued to increase until reaching equilibrium after 50 min. The results confirmed our hypothesis that a portion of the active ·OH can be transformed into relatively stable ·CHO species during the synthesis process (Scheme S2).

Interestingly, ·CHO and ·OH radicals have been detected as super stable in Cuf-TMB NPs from the ESR signal intensity *versus* time. Even after 52 days in ambient conditions, strong ESR signals of ·OH at 327 mT and ·CHO radicals at 323 mT (Figure 4C) were still detected. Only a ∼28.56% decrease in the intensity of the ·OH signal, while ∼9.84% increase in the intensity of the ·CHO signal after 18 days, suggesting a conversion of a portion of ·OH radicals to ·CHO. Although we could not completely rule out the possibility of ·OH species attenuation, it is likely minimal since the ·OH and ·CHO radicals were detectable for over 52 days. In contrast, ESR signals of these two radicals in purchased Fe_3_O_4_, CuO, Cu_2_O, and carbon NPs are much weaker compared to those observed in Cuf-TMB NPs (Figure S21). The superb stability of these radicals in Cuf-TMB NPs could be attributed to the steric hindrance effect of the TMB ligand and the stabilizing effect of the formate ligand, which will be thoroughly discussed in the following sections.^53^ The radical transformation was also detected upon the introduction of extra H_2_O_2_, which is a key step in the verification of POD-like activity. The signal of ·CHO was present at 6 min but disappeared by 18 min, while the signal of the ·OH radical was enhanced (Figure 4D). These results indicated that a controlled release of radicals can be achieved in the catalytic process.

The impact of TMB on the POD-like activity was certified by substituting TMB with TMB analogues with different steric hindrances during the synthesis (Figure S22). Compared with other Cuf-amine NPs, Cuf-TMB NPs showed much higher POD-like activity, irrespective of whether TMB or OPD was used as the substrate (Table S9). These results indicate the steric hindrance caused by the −CH_3_ group on the benzene ring can impede the access of oxidized species to reactive sites and thus stabilize the radicals.^54^ The influence of formate in stabilizing radicals of the Cuf-TMB system was studied by comparing the ESR spectra of Cuf-TMB with CuCl_2_-TMB and other traditional nanozymes (Figure S23). It was demonstrated that no ·CHO species can be captured in CuCl_2_-TMB, indicating the contribution of formate in transforming and stabilizing radicals. The above results suggest that the appropriate anion structure and steric hindrance effect can effectively promote the controlled release of free radicals. In addition, the influence of Cuf on the POD-like performances was investigated by designing Cu NPs coordinated with other ligands (amino acids, nucleotides, *etc.*) and comparing their performance with Cuf-TMB NPs. The as-prepared Cuf-TMB NPs showed the best POD-like performance (Figures S24–S26, Tables S10–S11) among the Cu-amine complexes. DFT calculations with a structure of Cu coordinated with two nitrogen and two oxygen atoms (CuN_2_O_2_) as a model for other Cu-amine complexes (Figure S27) revealed an uphill reaction energy (up to 0.86 eV) for the H transfer process between the two *OH species. This reaction energy was higher than the overall energy barrier in CuN_3_O, indicating the superior POD-like activity of Cuf-TMB NPs. Meanwhile, the zeta potential of Cuf-TMB NPs under pH 7 was measured to be 4.98 mV, which was favorable for the stability of radicals (Figure S28). These findings highlight that the Cuf-TMB NPs fabricated from TMB, H_2_O_2_, and Cuf can form a stable structure for ·CHO and ·OH radicals, thereby improving the catalytic activity.

### Application of Cuf-TMB NPs for Biosensing

Based on the excellent properties of Cuf-TMB NPs, a sensitive biosensing platform was developed as follows: The platform involved using agarose hydrogels as a visual assay platform to incorporate Cuf-TMB NPs, creating a visual detection test kit. The volume ratio of Cuf-TMB NP suspension to agarose hydrogel was optimized to be 8:2, as it showed the best catalytic properties (Figure S29). SEM and EDS mapping images indicated the successful loading of Cuf-TMB NPs into agarose hydrogels (Figures S30, S31). The designed test kit was utilized for the colorimetric detection of H_2_O_2_ by taking advantage of its efficient catalytic activity. The absorbance caused by the oxidized TMB increased proportionally with the concentration of H_2_O_2_. The concentration–response curve spanned from 0 to 10 mM, with a linear relationship in the range of 0–1 mM (*R*^2^ = 0.9963), and limited detection was as low as 0.1 μM (Figure S32).

**Figure 5:**
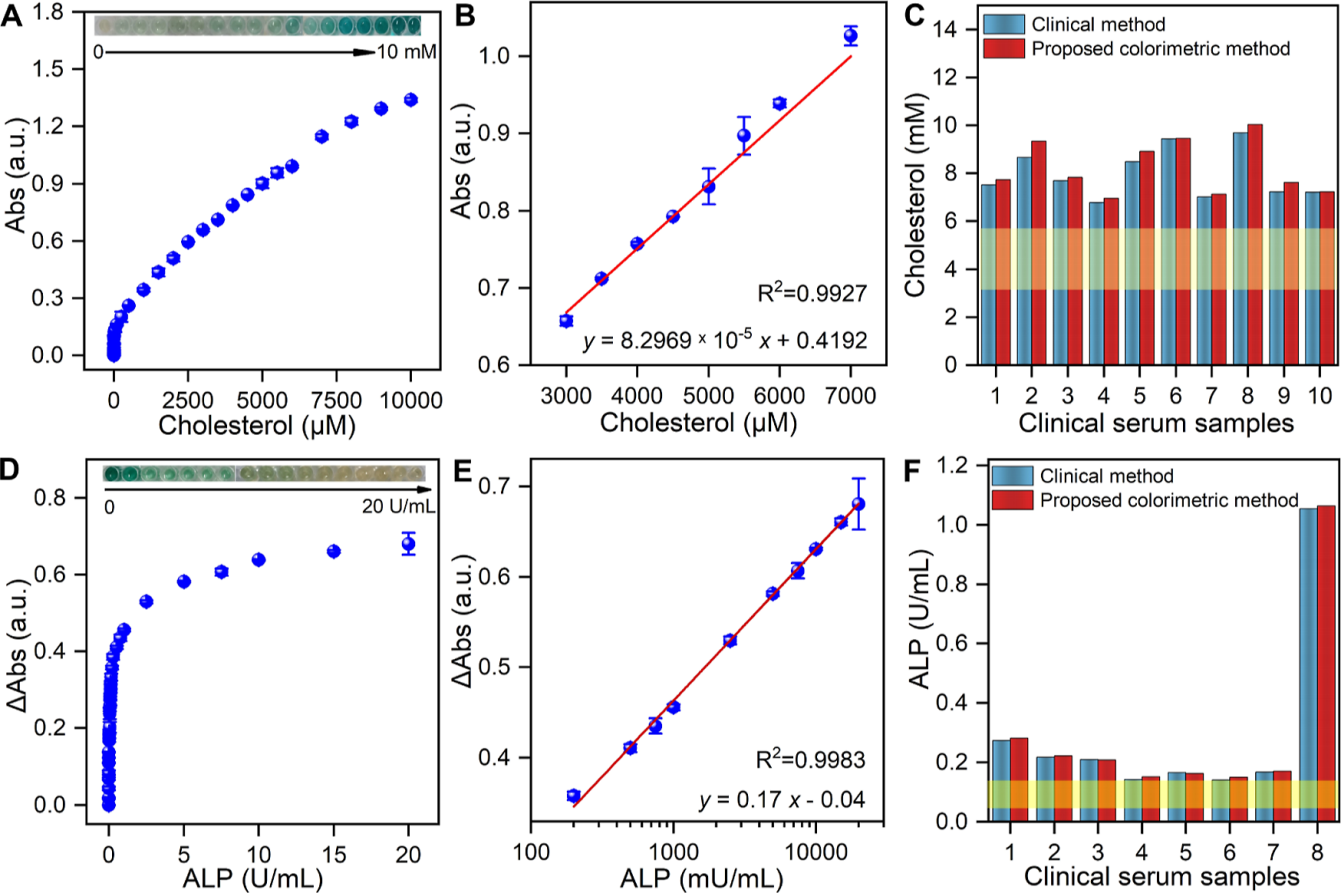
Application of Cuf-TMB NPs for biosensing. (A) Response curve of the cholesterol concentration from 0–10 mM. Inset: Photographs of oxidation products in the presence of cholesterol with different concentrations. (B) Calibration plot shows the linear relationship in the range of 3–7 mM. (C) Concentration of cholesterol in clinical serum samples was determined by proposed colorimetric methods and clinical CHOD-POD methods. Residual standard deviations (RSD) of NO.1–10 were 2.8, 5.1, 1.7, 2.5, 5.0, 0.3, 1.7, 3.6, 5.4, and 0.4% in order. (D) Response curve of the ALP concentration from 0–20 U mL^–1^. Inset: Photographs in the presence of ALP with different concentrations. (E) Linear range for ALP detection: 0.1–10 U mL^–1^; (F) Concentration of ALP in clinical serum samples determined by proposed colorimetric methods and clinical rate methods. RSD of no. 1–8 was 2.5, 1.9, 0.7, 2.4, 7.4, 1.4, and 0.9%. The blue cylinders indicated the clinical assay and the red cylinders reflected the colorimetric method used in this work; the yellow area represents the amount of cholesterol and ALP in healthy controls.

Moreover, the designed biosensor was used for the detection of biomolecules such as cholesterol, as H_2_O_2_ is an oxidation product of biomolecules such as glucose, cholesterol, *etc.* The detection of cholesterol was achieved in a one-step process by coupling the oxidation of cholesterol with the oxidation of H_2_O_2_, facilitated by Cuf-TMB NPs acting as nanozymes. Thus, the amount of cholesterol can be indirectly determined by monitoring the *in situ* production of H_2_O_2_ as a byproduct of the cholesterol oxidation reaction with the aid of ChoX. From the cholesterol response curve obtained at 652 nm, the absorbance of cholesterol exhibited a linear correlation with its concentrations from 0.1 to 100 μM (*R*^2^ = 0.9989), 1 to 4 mM (*R*^2^ = 0.9965), and 3 to 7 mM (*R*^2^ = 0.9927) (Figures 5A,B, S33). It should be noted that the detection limit of cholesterol was as low as 5 nM, which surpasses the sensitivity of other colorimetric biosensors and is even comparable to fluorometric or electrochemical methods (Table S12). To assess the practicability of this biosensor, the proposed method was applied to detect cholesterol in human serum samples by using the cholesterol oxidase-POD (CHOD-POD) method (see details in the experimental in the Supporting Information). As shown in Table S13, the recovery coefficient of all the samples was more than ∼95%. Cholesterol levels in patients with triglycerides were further measured with errors of less than 5% compared to standard values (Figure 5C).

Furthermore, ALP can hydrolyze AA2P to AA, which is a type of radical-breaker antioxidant and inhibits the oxidized reaction of TMB in agarose hydrogels containing Cuf-TMB NPs (Figures S34, S35). The detection of ALP was also achieved in a one-step process by coupling the hydrolysis reaction of AA2P with the oxidation of H_2_O_2_ by Cuf-TMB NPs as nanozymes (Figure 5D,E). The linearity range for ALP was 10^–6^ to 10 mU mL^–1^ (*R*^2^ = 0.9965), 50–125 mU mL^–1^ (*R*^2^ = 0.9972), and 0.2–20 mU mL^–1^ (*R*^2^ = 0.9983, Figure S35). Notably, the lower limit of detection was as low as 10^–5^ mU mL^–1^. The sensitivity of the Cuf-TMB NP-based colorimetric method surpassed that of other reports (Table S14). Furthermore, the practical application of the biosensor was demonstrated by analyzing the ALP level in serum samples. As shown in Table S15, the ALP amount was successfully detected with ∼95% accuracy, and RSD was less than 10% compared to the clinical rate method (Figure 5F). Additionally, the Cuf-TMB-based biosensor showed its ability to sensitively detect other biomolecules, such as Cys and glucose, *etc.* (Figures S36, S37, Table S16). These results indicate that the Cuf-TMB NP-based biosensor can be applied to determine ALP and cholesterol in real samples, offering promising applications in biological and biomedical clinics (Table S17).

## Conclusions

In summary, Cu-amine nanozymes with different coordination structures (e.g., Cuf-TMB) were unexpectedly prepared by the pre-catalytic strategy and displayed excellent performance. The as-prepared Cuf-TMB nanozymes show comparable catalytic activity (*K*_m_, 1.02 × 10^–5^ mM^–1^; *K*_cat_, 3.09 × 10^–2^ s^–1^) and kinetics to the natural enzyme POD toward H_2_O_2_ decomposition. The superb catalytic activity is attributed to the low energy of the oxidation process of POD substrates by *O intermediate species generated from *OH species from DFT calculations. More importantly, the reversibility and transformability of ·CHO and ·OH species were captured in the Cuf-TMB nanozyme system by *in situ* ESR measurements. The controlled release of radicals can be achieved by introducing amine ligands or H_2_O_2_ as an agent. These radicals exhibited remarkable stability for up to 52 days, owing to the steric hindrance effect of the TMB ligand and the stabilizing effect of the formate ligand. Cuf-TMB nanozymes enabled the sensitive detection of various analytes such as cholesterol, ALP, glucose, AA, and Cys. This discovery could inspire new ideas for understanding the radical-related mechanism of nanozymes and hold potential in catalysis, biological applications, and environmental monitoring and protection.

## Supporting Information Available

Synthesis and characterizations of Cuf-TMB (XRD, SEM, TEM, HRTEM, FT-IR, Raman, and XPS); comparison of POD-like activities of CuX-TMB and different nanozymes; Michaelis–Menten kinetics of nanozymes and HRP; ESR spectra of CuX-TMB; DFT calculations; catalytic activity of Cuf-amine; applications of Cuf-TMB; and the detection of cholesterol, AA, ALP, Cys, and glucose (PDF)

## Notes

All data needed to evaluate the conclusions in the paper are present in the paper and/or the Supporting Information.

## References

[ref1] Wei H.; Wang E. Nanomaterials with Enzyme-like Characteristics (Nanozymes): Next-Generation Artificial Enzymes. Chem. Soc. Rev. 2013, 42, 6060–6093. 10.1039/c3cs35486e. 23740388

[ref2] Jiang D.; Ni D.; Rosenkrans Z. T.; Huang P.; Yan X.; Cai W. Nanozyme: New Horizons for Responsive Biomedical Applications. Chem. Soc. Rev. 2019, 48, 3683–3704. 10.1039/c8cs00718g. 31119258 PMC6696937

[ref3] Wang H.; Wan K.; Shi X. Recent Advances in Nanozyme Research. Adv. Mater. 2019, 31, e1805368 10.1002/adma.201805368. 30589120

[ref4] Robert A.; Meunier B. How to Define a Nanozyme. ACS Nano 2022, 16, 6956–6959. 10.1021/acsnano.2c02966. 35521947

[ref5] Ghosh S.; Roy P.; Karmodak N.; Jemmis E. D.; Mugesh G. Nanoisozymes: Crystal-Facet-Dependent Enzyme-Mimetic Activity of V_2_O_5_ Nanomaterials. Angew. Chem., Int. Ed. 2018, 57, 4510–4515. 10.1002/anie.201800681. 29424075

[ref6] Li S.; Shang L.; Xu B.; Wang S.; Gu K.; Wu Q.; Sun Y.; Zhang Q.; Yang H.; Zhang F.; Gu L.; Zhang T.; Liu H. A Nanozyme with Photo-Enhanced Dual Enzyme-Like Activities for Deep Pancreatic Cancer Therapy. Angew. Chem. 2019, 131, 12754–12761. 10.1002/ange.201904751. 31237404

[ref7] Singh N.; Mugesh G. CeVO_4_ Nanozymes Catalyze the Reduction of Dioxygen to Water without Releasing Partially Reduced Oxygen Species. Angew. Chem., Int. Ed. 2019, 58, 7797–7801. 10.1002/anie.201903427. 30950157

[ref8] Wei H.; Gao L.; Fan K.; Liu J.; He J.; Qu X.; Dong S.; Wang E.; Yan X. Nanozymes: A Clear Definition with Fuzzy Edges. Nano Today 2021, 40, 101269 10.1016/j.nantod.2021.101269.

[ref9] Zhang R.; Yan X.; Fan K. Nanozymes Inspired by Natural Enzymes. Acc. Mater. Res. 2021, 2, 534–547. 10.1021/accountsmr.1c00074.

[ref10] Gao L.; Zhuang J.; Nie L.; Zhang J.; Zhang Y.; Gu N.; Wang T.; Feng J.; Yang D.; Perrett S.; Yan X. Intrinsic Peroxidase-Like activity of Ferromagnetic Nanoparticles. Nat. Nanotechnol. 2007, 2, 577–583. 10.1038/nnano.2007.260. 18654371

[ref11] Xi Z.; Cheng X.; Gao Z.; Wang M.; Cai T.; Muzzio M.; Davidson E.; Chen O.; Jung Y.; Sun S.; Xu Y.; Xia X. Strain Effect in Palladium Nanostructures as Nanozymes. Nano Lett. 2020, 20, 272–277. 10.1021/acs.nanolett.9b03782. 31821008

[ref12] Czescik J.; Zamolo S.; Darbre T.; Rigo R.; Sissi C.; Pecina A.; Riccardi L.; De Vivo M.; Mancin F.; Scrimin P. A Gold Nanoparticle Nanonuclease Relying on a Zn(II) Mononuclear Complex. Angew. Chem., Int. Ed. 2021, 60, 1423–1432. 10.1002/anie.202012513. PMC7839518 32985766

[ref13] Liu M.; Zhang S.; Wang Y.; Liu J.; Hu W.; Lu X. Hexavalent Chromium as a Smart Switch for Peroxidase-Like Activity Regulation *Via* the Surface Electronic Redistribution of Silver Nanoparticles Anchored on Carbon Spheres. Anal. Chem. 2022, 94, 1669–1677. 10.1021/acs.analchem.1c04219. 35020355

[ref14] Dong H.; Du W.; Dong J.; Che R.; Kong F.; Cheng W.; Ma M.; Gu N.; Zhang Y. Depletable Peroxidase-Like Activity of Fe_3_O_4_ Nanozymes Accompanied with Separate Migration of Electrons and Iron Ions. Nat. Commun. 2022, 13, 5365 10.1038/s41467-022-33098-y. 36097172 PMC9467987

[ref15] Fu S.; Chen H.; Yang W.; Xia X.; Zhao S.; Xu X.; Ai P.; Cai Q.; Li X.; Wang Y.; Zhu J.; Zhang B.; Zheng J. C. ROS-Targeted Depression Therapy *via* BSA-Incubated Ceria Nanoclusters. Nano Lett. 2022, 22, 4519–4527. 10.1021/acs.nanolett.2c01334. 35583518 PMC9185743

[ref16] Peng J.; Weng J. Enhanced Peroxidase-Like activity of MoS_2_/Graphene Oxide Hybrid with Light Irradiation for Glucose Detection. Biosens. Bioelectron. 2017, 89, 652–658. 10.1016/j.bios.2015.12.034. 26711356

[ref17] Song Y.; Qu K.; Zhao C.; Ren J.; Qu X. Graphene Oxide: Intrinsic Peroxidase Catalytic Activity and Its Application to Glucose Detection. Adv. Mater. 2010, 22, 2206–2210. 10.1002/adma.200903783. 20564257

[ref18] Huang Y.; Chen B.; Duan J.; Yang F.; Wang T.; Wang Z.; Yang W.; Hu C.; Luo W.; Huang Y. Graphitic Carbon Nitride (g-C_3_N_4_): An Interface Enabler for Solid-State Lithium Metal Batteries. Angew. Chem., Int. Ed. 2020, 59, 3699–3704. 10.1002/anie.201914417. 31851408

[ref19] Ji S.; Jiang B.; Hao H.; Chen Y.; Dong J.; Mao Y.; Zhang Z.; Gao R.; Chen W.; Zhang R.; Liang Q.; Li H.; Liu S.; Wang Y.; Zhang Q.; Gu L.; Duan D.; Liang M.; Wang D.; Yan X.; Li Y. Matching the Kinetics of Natural Enzymes with a Single-Atom Iron Nanozyme. Nat. Catal. 2021, 4, 407–417. 10.1038/s41929-021-00609-x.

[ref20] Xi J.; Wei G.; An L.; Xu Z.; Xu Z.; Fan L.; Gao L. Copper/Carbon Hybrid Nanozyme: Tuning Catalytic Activity by the Copper State for Antibacterial Therapy. Nano Lett. 2019, 19, 7645–7654. 10.1021/acs.nanolett.9b02242. 31580681

[ref21] Wang Q.; Cheng C.; Zhao S.; Liu Q.; Zhang Y.; Liu W.; Zhao X.; Zhang H.; Pu J.; Zhang S.; Zhang H.; Du Y.; Wei H. A Valence-Engineered Self-Cascading Antioxidant Nanozyme for the Therapy of Inflammatory Bowel Disease. Angew. Chem., Int. Ed. 2022, 61, e202201101 10.1002/anie.202201101. 35452169

[ref22] Hong Q.; Yang H.; Fang Y.; Li W.; Zhu C.; Wang Z.; Liang S.; Cao X.; Zhou Z.; Shen Y.; Liu S.; Zhang Y. Adaptable Graphitic C_6_N_6_-Based Copper Single-Atom Catalyst for Intelligent Biosensing. Nat. Commun. 2023, 14, 2780 10.1038/s41467-023-38459-9. 37188673 PMC10185664

[ref23] Lin L. S.; Huang T.; Song J.; Ou X. Y.; Wang Z.; Deng H.; Tian R.; Liu Y.; Wang J. F.; Liu Y.; Yu G.; Zhou Z.; Wang S.; Niu G.; Yang H. H.; Chen X. Synthesis of Copper Peroxide Nanodots for H_2_O_2_ Self-Supplying Chemodynamic Therapy. J. Am. Chem. Soc. 2019, 141, 9937–9945. 10.1021/jacs.9b03457. 31199131

[ref24] Jiao L.; Yan H.; Wu Y.; Gu W.; Zhu C.; Du D.; Lin Y. When Nanozymes Meet Single-Atom Catalysis. Angew. Chem., Int. Ed. 2020, 59, 2565–2576. 10.1002/anie.201905645. 31209985

[ref25] Wan K.; Jiang B.; Tan T.; Wang H.; Liang M. Surface-Mediated Production of Complexed *OH Radicals and Fe=O Species as a Mechanism for Iron Oxide Peroxidase-Like Nanozymes. Small 2022, 18, 2204372 10.1002/smll.202204372. 36316230

[ref26] Xu Y.; Zhou Z.; Deng N.; Fu K.; Zhu C.; Hong Q.; Shen Y.; Liu S.; Zhang Y. Molecular Insights of Nanozymes from Design to Catalytic Mechanism. Sci. Chin. Chem. 2023, 66, 1318–1335. 10.1007/s11426-022-1529-y. PMC9923663 36817323

[ref27] Chen F.; Liu L. L.; Wu J. H.; Rui X. H.; Chen J. J.; Yu Y. Single-Atom Iron Anchored Tubular g-C_3_N_4_ Catalysts for Ultrafast Fenton-Like Reaction: Roles of High-Valency Iron-Oxo Species and Organic Radicals. Adv. Mater. 2022, 34, e2202891 10.1002/adma.202202891. 35679161

[ref28] Zhou Z.; Wang Y.; Peng F.; Meng F.; Zha J.; Ma L.; Du Y.; Peng N.; Ma L.; Zhang Q.; Gu L.; Yin W.; Gu Z.; Tan C. Intercalation-Activated Layered MoO_3_ Nanobelts as Biodegradable Nanozymes for Tumor-Specific Photo-Enhanced Catalytic Therapy. Angew. Chem., Int. Ed. 2022, 61, e202115939 10.1002/anie.202115939. 35080098

[ref29] Zandieh M.; Liu J. Surface Science of Nanozymes and Defining a Nanozyme Unit. Langmuir 2022, 38, 3617–3622. 10.1021/acs.langmuir.2c00070. 35290071

[ref30] Huang Y.; Ren J.; Qu X. Nanozymes: Classification, Catalytic Mechanisms, Activity Regulation, and Applications. Chem. Rev. 2019, 119, 4357–4412. 10.1021/acs.chemrev.8b00672. 30801188

[ref31] Wang M.; Chang M.; Li C.; Chen Q.; Hou Z.; Xing B.; Lin J. Tumor-Microenvironment-Activated Reactive Oxygen Species Amplifier for Enzymatic Cascade Cancer Starvation/Chemodynamic/Immunotherapy. Adv. Mater. 2022, 34, e2106010 10.1002/adma.202106010. 34699627

[ref32] Singh N.; Sherin G. R.; Mugesh G. Antioxidant and Prooxidant Nanozymes: From Cellular Redox Regulation to Next-Generation Therapeutics. Angew. Chem., Int. Ed. 2023, 62, e202301232 10.1002/anie.202301232. 37083312

[ref33] Wei H.; Wang E. Fe_3_O_4_ Magnetic Nanoparticles as Peroxidase Mimetics and Their Applications in H_2_O_2_ and Glucose Detection. Anal. Chem. 2008, 80, 2250–2254. 10.1021/ac702203f. 18290671

[ref34] Sun J.; Xia F.; Zhang S.; Zhang B.; Guan Y.; Hu X.; Xue P.; Yang S.; Zhou Y.; Ling D.; Li F. A Selective Nano Cell Cycle Checkpoint Inhibitor Overcomes Leukemia Chemoresistance. Small 2023, 19, 2300736 10.1002/smll.202300736. 37029565

[ref35] Rodríguez-López J. N.; Lowe D. J.; Hernández-Ruiz J.; Hiner A. N.; García-Cánovas F.; Thorneley R. N. Mechanism of Reaction of Hydrogen Peroxide with Horseradish Peroxidase: Identification of Intermediates in the Catalytic Cycle. J. Am. Chem. Soc. 2001, 123, 11838–11847. 10.1021/ja011853+. 11724589

[ref36] Cai W.; Chen F.; Shen X.; Chen L.; Zhang J. Enhanced Catalytic Degradation of AO7 in the CeO_2_-H_2_O_2_ System with Fe^3+^ Doping. Appl. Catal., B 2010, 101, 160–168. 10.1016/j.apcatb.2010.09.031.

[ref37] Zhang X.; Li G.; Chen G.; Wu D.; Zhou X.; Wu Y. Single-Atom Nanozymes: A Rising Star for Biosensing and Biomedicine. Coordin. Chem. Rev. 2020, 418, 213376 10.1016/j.ccr.2020.213376.

[ref38] Kresse G.; Furthmüller J. Efficiency of Ab-Initio Total Energy Calculations for Metals and Semiconductors Using a Plane-Wave Basis Set. Comput. Mater. Sci. 1996, 6, 15–50. 10.1016/0927-0256(96)00008-0. 9984901

[ref39] Kresse G.; Furthmüller J. Efficient Iterative Schemes for Ab-Initio Total-Energy Calculations Using a Plane-Wave Basis Set. Phys. Rev. B 1996, 54, 11169–11186. 10.1103/physrevb.54.11169. 9984901

[ref40] Blochl P. E. Projector Augmented-Wave Method. Phys. Rev. 1994, 50, 17953–17979. 10.1103/physrevb.50.17953. 9976227

[ref41] Perdew J. P.; Burke K.; Ernzerhof M. Generalized Gradient Approximation Made Simple. Phys. Rev. Lett. 1996, 77, 3865–3868. 10.1103/physrevlett.77.3865. 10062328

[ref42] Grimme S.; Antony J.; Ehrlich S.; Krieg H. A Consistent and Accurate Ab-Initio Parametrization of Density Functional Dispersion Correction (DFT-D) for the 94 Elements H-Pu. J. Chem. Phys. 2010, 132, 154104 10.1063/1.3382344. 20423165

[ref43] Press W. H.; Teukolsky S. A.; Vetterling W. T.; Flannery B. P. Numerical Recipes in C, The Art of Scientific Computing Second Edition; Cambridge University Press: New York, 1988.

[ref44] Nocedal J. Updating Quasi-Newton Matrices with limited Storage. Math. Comput. 1980, 35, 773–782. 10.1090/s0025-5718-1980-0572855-7.

[ref45] Shanno D. F. Conditioning of Quasi-Newton Methods for Function Minimization. Math. Comput. 1970, 24, 647–656. 10.1090/s0025-5718-1970-0274029-x.

[ref46] Henkelman G.; Uberuaga B. P.; Jónsson H. A Climbing Image Nudged Elastic Band Method for Finding Saddle Points and Minimum Energy Paths. J. Chem. Phys. 2000, 113, 9901–9904. 10.1063/1.1329672.

[ref47] Sheppard D.; Henkelman G. Paths to Which the Nudged Elastic Band Converges. J. Comput. Chem. 2011, 32, 1769–1771. 10.1002/jcc.21748. 21328409

[ref48] Momma K.; Izumi F. *VESTA 3* for three-dimensional visualization of crystal, volumetric and morphology data. J. Appl. Crystallogr. 2011, 44, 1272–1276. 10.1107/s0021889811038970.

[ref49] Allain C. C.; Poon L. S.; Chan C. S. G.; Richmond W.; Fu P. C. Enzymatic Determination of Total Serum Cholesterol. Clin. Chem. 1974, 20, 470–475. 10.1093/clinchem/20.4.470. 4818200

[ref50] Salter R. S.; Fitchen J.; Bain B.; Bella M.; Bergman S.; Biotelle A. C.; Bulthaus M.; Butterworth F.; Collins P.; Davag R.; et al. Evaluation of a Chemiluminescence Method for Measuring Alkaline Phosphatase Activity in Whole Milk of Multiple Species and Bovine Dairy Drinks: Interlaboratory Study. J. AOAC Int. 2006, 89, 1061–1070. 10.1093/jaoac/89.4.1061. 16915846

[ref51] Zhang S.; Li Y.; Sun S.; Liu L.; Mu X.; Liu S.; Jiao M.; Chen X.; Chen K.; Ma H.; Li T.; Liu X.; Wang H.; Zhang J.; Yang J.; Zhang X. D. Single-Atom Nanozymes Catalytically Surpassing Naturally Occurring Enzymes as Sustained Stitching for Brain Trauma. Nat. Commun. 2022, 13, 4744 10.1038/s41467-022-32411-z. 35961961 PMC9374753

[ref52] Bauer N. A.; Hoque E.; Wolf M.; Kleigrewe K.; Hofmann T. Detection of the Formyl Radical by EPR Spin-Trapping and Mass Spectrometry. Free Radical Biol. Med. 2018, 116, 129–133. 10.1016/j.freeradbiomed.2018.01.002. 29307725

[ref53] Peng J.; Chen B.; Wang Z.; Guo J.; Wu B.; Hao S.; Zhang Q.; Gu L.; Zhou Q.; Liu Z.; Hong S.; You S.; Fu A.; Shi Z.; Xie H.; Cao D.; Lin C. J.; Fu G.; Zheng L. S.; Jiang Y.; Zheng N. Surface Coordination Layer Passivates Oxidation of Copper. Nature 2020, 586, 390–394. 10.1038/s41586-020-2783-x. 33057223

[ref54] Jegerschoeld C.; Arellano J. B.; Schroeder W. P.; van Kan P. J.; Baron M.; Styring S. Copper(II) Inhibition of Electron Transfer Through Photosystem II Studied by EPR Spectroscopy. Biochemistry 1995, 34, 12747–12754. 10.1021/bi00039a034. 7548028

