# Supplementary material for: Stabilized and Controlled Release of Radicals within Copper Formate-Based Nanozymes for Biosensing
Source: ACS Appl Mater Interfaces. 2023 Sep 7;15(37):43431–40. doi: 10.1021/acsami.3c08326 (PMC10520911; doi:10.1021/acsami.3c08326)
Supplement: Supplementary file 1 — am3c08326_si_001.pdf [file am3c08326_si_001.pdf]

Supporting Information

Stabilized and controlled release of radicals within copper formate-based nanozymes for biosensing

Yue Zhou[1], Xiaohua Chen[2], Shaoqi Zhan[3]*, Qiang Wang[4], Feng Deng[4], Qingzhi Wu[1], Jian Peng[1]*

[1]State Key Laboratory of Advanced Technology for Materials Synthesis and Processing, and School of Chemistry, Chemical Engineering and Life Science, Wuhan University of Technology, Wuhan 430070, China

[2]Department of Laboratory Medicine, General Hospital of Central Theater Command, Wuhan 430070, China

[3]Department of Chemistry – BMC, Uppsala University, BMC Box 576, S-751 23 Uppsala, Sweden

[4]State Key Laboratory of Magnetic Resonance and Atomic and Molecular Physics, Innovation Academy for Precision Measurement Science and Technology, Chinese Academy of Sciences, Wuhan, 430071, China

E. mail: jianpeng@whut.edu.cn; shaoqi.zhan@chem.ox.ac.uk

Experimental Section

Synthesis of CuX-TMB NPs. For the synthesis of CuX-TMB NPs, the parameters were the same as those of Cuf-TMB NPs, except that the Cuf was replaced by other copper salts (denoted as CuX).

Synthesis of Cuf-amine NPs. For the synthesis of Cuf-amine NPs, the parameters were the same as those of Cuf-TMB NPs, except that the TMB was replaced by other amine ligands.

Synthesis of Cuf-amino acids NPs. For the synthesis of Cuf-amino acids NPs, the parameters were the same as those of Cuf-TMB NPs, except for the TMB ligand which was replaced by other amino acids (denoted as Cuf-amino acids NPs).

Synthesis of Cuf-nucleotides NPs. For the synthesis of Cuf-nucleotides NPs, the parameters were the same as those of Cuf-TMB NPs, except for the TMB ligand which was replaced by nucleotides (denoted as Cuf-nucleotides NPs).

Cyclic Voltammetry. Cyclic voltammograms were recorded from −1 V to +2 V at scan rates of 50 mV/s at room temperature. Au, Ag/AgCl and platinum wire were used as the working, reference and counter electrodes, respectively. The Au electrode was carefully polished with 50 nm alumina slurry successively and then ultrasonicated in water for a few minutes. Typically, as-prepared Cuf-TMB NPs suspension was used as the supporting electrolyte.

POD-like activity of Cuf-TMB NPs.

The Fenton mechanism is presented by equation 1,

$$2\,Fe^{2+} + 2H_2O_2 \longrightarrow 2\,Fe^{3+} + \cdot 2OH + 2\,OH^- \qquad (1)$$

POD-like activity was exhibited by equation 2,

$$\mathrm{TMB} + H_2O_2 \xrightarrow{\text{Peroxidase(Cuf-TMB NPs)}} \mathrm{OxTMB} + H_2O \qquad (2)$$

The Michaelis-Menten constant was calculated with equation 3,4,

$$V_0 = V_{max} \frac{[s]}{[s] + K_m} \qquad (3)$$

$$\frac{1}{V_0} = \frac{1}{V_{max}} + \frac{K_m}{V_{max}} \frac{1}{[s]} \qquad (4)$$

where K_m is the Michaelis constant, V_{max} is the maximum reaction rate when the enzyme is saturated with the substrate, [S] is the substrate concentration. For the kinetic parameter of traditional nanozymes, the testing parameters were the same as those of Cuf-TMB NPs, except that the Cuf-TMB NPs were replaced by Fe_3O_4, C, CuO, Cu_2O, and Au NPs.

Identification of radicals. The radical trapping reaction was displayed with equation 5,

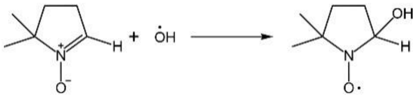

(5)

Here, 5,5-dimethyl-1-pyrroline-n-oxide (DMPO) was a trapping agent, which will trap ·OH and show the ESR signal.

ESR spectra of traditional nanozymes. Traditional nanozymes (Fe_3O_4 NPs, C NPs, CuO NPs, Cu_2O NPs, and Au NPs) were mixed with H_2O_2 (10 mM) and DMPO (1%) before the ESR spectra were collected.

H_2O_2 detection. 50 µL of H_2O_2 (0-10 mM) and 50 µL of TMB (2 mM) were injected into 96-well plates with 200 µL Cuf-TMB NPs hydrogels before the absorbance of 652 nm with time was collected by a microplate reader.

Glucose detection. 450 μL of glucose with different concentrations and 50 μL glucose oxidase solution (1 mg/mL) were mixed in a 1.5 mL centrifuge tube, which was kept at 37 °C for 20 min under shaking. the mixture, together with 50 μL of TMB (2 mM), was injected into 96-well plates with 200 μL Cuf-TMB NPs hydrogels before the absorbance of 652 nm with time was recorded by a microplate reader. To evaluate the specificity for glucose detection, glucose was replaced with 5 mM of fructose, lactose, maltose, and dopamine during the measurement while keeping other conditions the same as above.

Total cholesterol detection in human serum. Cholesterol in serum includes free cholesterol and cholesterol ester. Cholesterol esters were hydrolyzed into cholesterol with the cholesterol esterase (ChE). Total cholesterol detection in serum was carried out as follows: (1) The human serum samples were prediluted 150-fold with 5% aqueous Triton X-100 solution. (2) The serum samples were further incubated with ChE (1 mU/mL) and Chox (5 mg/mL) at 37 °C for 30 min. (3) 200 μL of Cuf-TMB NPs hydrogel, 50 μL of the incubated solution, 50 μL of H_2O_2 (10 mM), and 50 μL of TMB (2 mM) were added into a 96-well plate in turn. (4) The absorbance was detected as described above.

ALP Detection in Human Serum. The determination of ALP in serum was operated as follows: (1) 100 μL of serum was diluted to 900 μL using PBS buffer solution. (2) 100 μL of AA2P (100 mM), 100 μL of different serum solution, and 50 μL of Tris-HCl (pH 8.0, 50 mM) were added into the tube and incubated at 37 °C for 40 min under shaking. (3) 200 μL of Cuf-TMB NPs hydrogel, 50 μL of an incubated solution, 50 μL of H_2O_2 (10 mM), and 50 μL of TMB (2 mM) were added into a 96-well plate in turn. (4) The absorbance was detected as described above.

Rate method. Concentration of ALP was calculated with equation 6,

$$\text{ALP(U/L)} = \Delta A / \text{min} \times {}^{\circ}F \qquad (6)$$

Where ΔA/m in is the absorbance change rate per minute, and °F is Fahrenheit.

Cysteine detection. 50 μL H_2O_2 (10 mM) and 50 μL TMB (2 mM) were added to the Cuf-TMB hydrogel when the hydrogel turned blue. Then, 50 μL of cysteine of different concentrations was dropped into the blue hydrogel before the absorbance (652 nm) at different intervals was recorded. 1 mM AA, 10 mM histidine, leucine, alanine, phenylalanine, threonine, valine, glycine, NaCl, and KCl solutions were used to evaluate the selectivity and anti-interference of the biosensor.

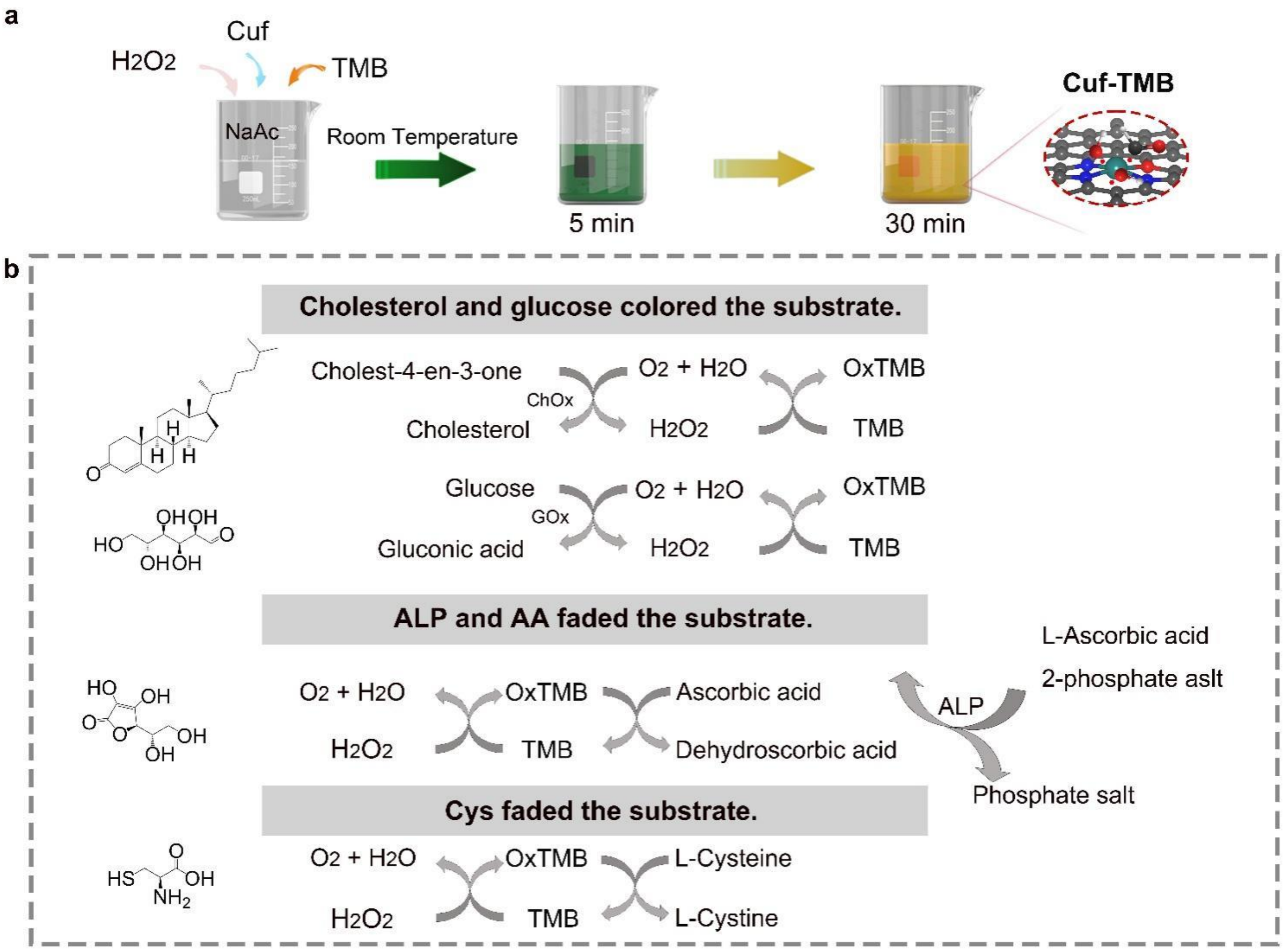

Scheme S1. (a) Schematic illustration of the fabrication process of the Cuf-TMB nanozyme; (b) Versatile applications of the Cuf-TMB nanozyme in cholesterol and glucose detection in serum, ALP detection in serum, and Cys determination.

The generation of ·CHO in the Cuf-TMB system was proposed to follow the steps:

$$Cu(HCOO)_2 + 2H_2O \leftrightarrow 2HCOOH + Cu(OH)_2 \quad (7)$$

$$HCOOH \leftrightarrow H^+ + HCOO^- \quad (8)$$

$$HCOO^- \overset{hv}{\leftrightarrow} \cdot CHO + O^- \quad (9$$

$$H_2O_2 \overset{Cu^{2+}}{\longleftrightarrow} 2 \cdot OH \quad (10)$$

Scheme S2: Cuf undergoes rapid hydrolysis, resulting in the formation of formic acid (HCOOH) and $Cu(OH)_2$ (Eq. 7). There is a decrease in pH from 7 to 5.8 (Figure S1), after adding H_2O_2 to the NaAc buffer. The weakly acidic environment provides H^+ to $HCOO^-$, enabling the stabilization of HCOOH formed by Cuf hydrolysis (Eq. 8). $HCOO^-$ can be decomposed to ·CHO and O^- in the presence of light (Eq. 9).[1-4] In the presence of Cu^{2+}, H_2O_2 can perform a Fenton-like reaction to produce the key intermediate ·OH which has been observed experimentally (Eq. 10).

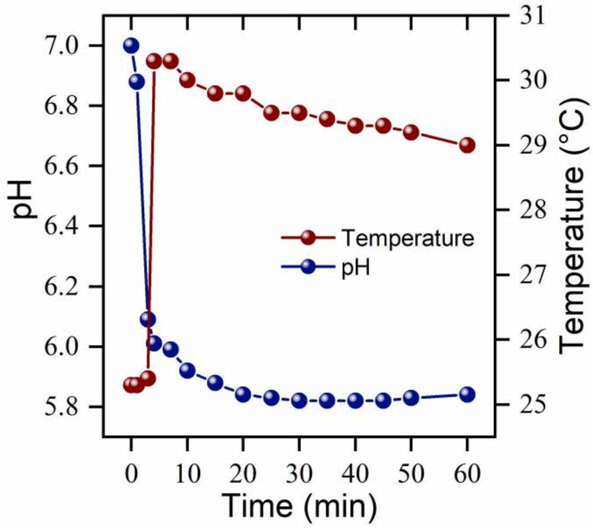

Figure S1. *In situ* monitoring of the pH and temperature change during the synthesis of Cuf-TMB NPs. *In situ* monitoring of the change of pH and temperature during the synthesis of Cuf-TMB NPs in Figure S12 showed the temperature increased obviously with decreased pH values upon the addition of TMB, indicating the synthesis of Cuf-TMB NPs was driven by the coordination of TMB to Cu(II).

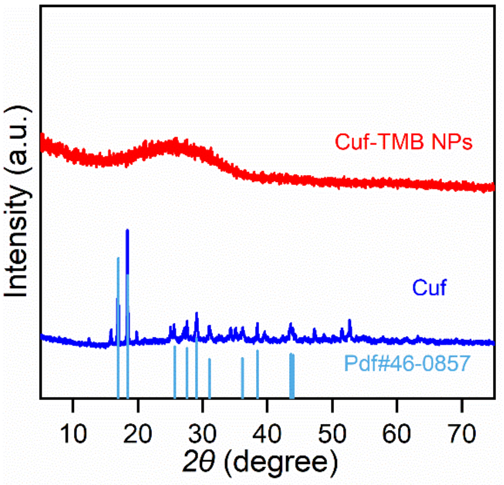

Figure S2. XRD pattern of as-prepared Cuf-TMB NPs. It is demonstrated that Cuf-TMB NPs were an amorphous structure. We observe that Cuf consists of very clear and sharp peaks, in contrast to Cuf-TMB, which is distinctly amorphous. This result demonstrates that the crystalline phase within Cuf-TMB may not have fully grown or reached the threshold for crystallization.

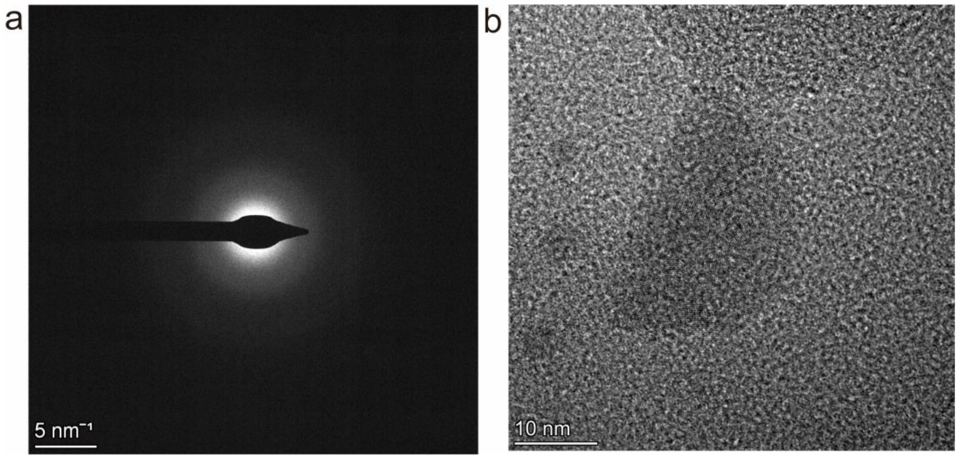

Figure S3. (a) The SAED pattern of Cuf-TMB NPs. SAED shows an amorphous dispersion ring, which proves that Cuf-TMB is an amorphous material. (b) HRTEM image of Cuf-TMB NPs. In the SAED experiment, we did not observe any obvious diffraction rings, while in the HAADF-TEM observation, no lattice bond distances were observed. These data provide support for the amorphous structure of Cuf-TMB.

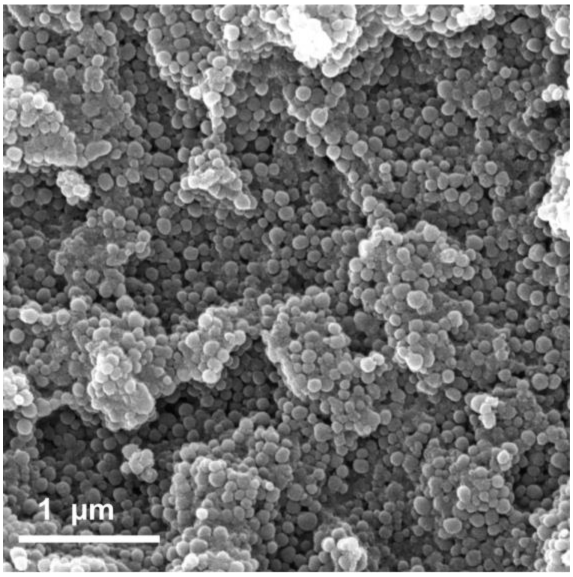

Figure S4. SEM image of Cuf-TMB NPs. Scale bar, 1 μm**.** From the SEM data, we observed that Cuf-TMB was in the form of small spheres with smooth surface and uniform distribution.

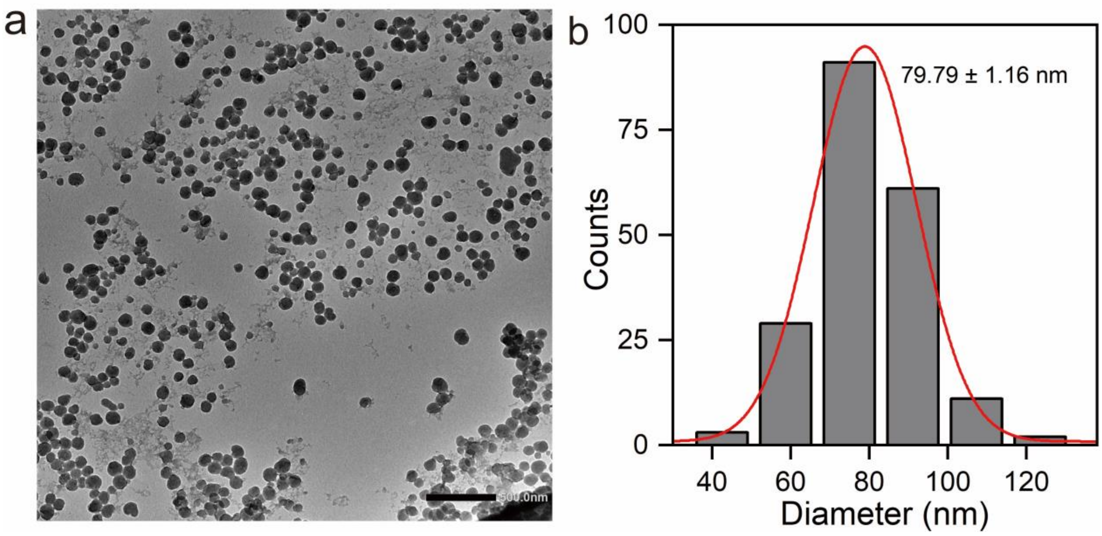

Figure S5. (a) TEM image of Cuf-TMB NPs. Scale bar, 500 nm. (b) Size distributions of Cuf-TMB NPs. TEM further demonstrated that Cuf-TMB was spherical. In addition, particle size statistics were performed and Cuf-TMB was found to have a particle size of about 79 nm.

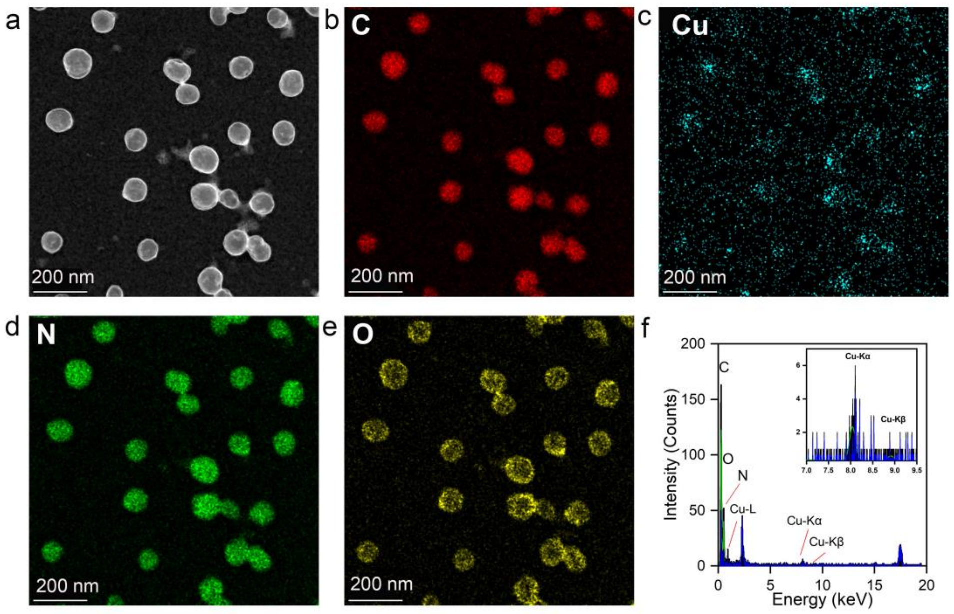

Figure S6. HAADF-STEM image (a) and corresponding energy dispersive X-ray spectroscopy (EDS) mapping images (b-e) of Cuf-TMB NPs. Scale bar, 200 nm. Color legend: (b) red, C; (c) blue, Cu; (d) green, N; (e) yellow, O. (f) EDS spectrum of Cuf-TMB NPs. For EDS mapping analysis, a single polyhedron was chosen. The Cu metal atoms as well as the elements C and N are uniformly distributed throughout the nanostructures. The element O is dispersed at the edges of the spheres. This provides more convincing support for the amorphous nature of the Cuf-TMB NPs.

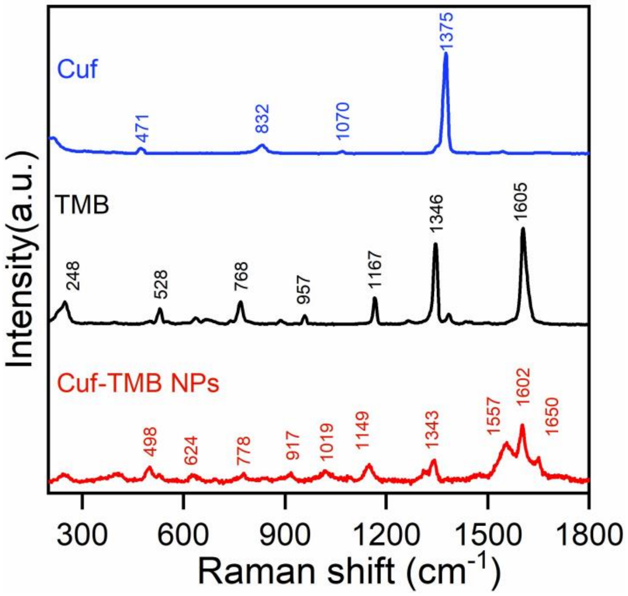

Figure S7. Raman spectra of Cuf, TMB, and Cuf-TMB NPs. The Raman spectrum of copper formate exhibited several characteristic peaks, at 1375, 1070, 832 and 471 cm^{-1}, representing ν_s (OCO), ρ (CH), ν_s (OCO) and ν_s (Cu-O), respectively.[5-9] Raman spectra of Cuf-TMB demonstrated a red-shift to 1602, 1343, 1149, 1019, 917, and 778 cm^{-1} compared to TMB, indicating the successful conjugation of formate and TMB ligand (Table S1).

Table S1. Analysis of different peaks from the Raman spectra of Cuf-TMB NPs including peaks, corresponding groups and references.

Raman shift (cm^{-1})	Functional group	Ref.
1650, 1557	C=O stretching	[10]
1602	ν ring + δ (CH)	[11]
1343	ν_s N-ring	[12]
1149	ρ (CH_3)	[12]
1019	ρ (CH_3)	[12]
917	ν_s of C-N	[13]
778, 625, 498	Benzene ring vibration	[12]

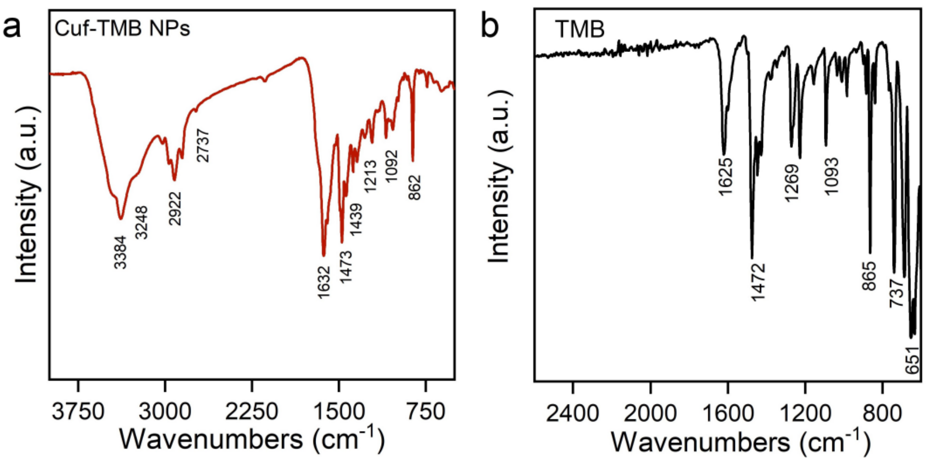

Figure S8. (a) FT-IR spectrum of Cuf-TMB NPs. (b) The FT-IR spectra of TMB. Compared to the TMB, Cuf-TMB showed obvious differences in spectrum peaks, at 3384, 2922, 3248, and 2737 cm^{-1} which attributed to O-H stretching and N-H vibration. Meanwhile, compared with TMB, the peaks at 651 and 737 cm^{-1} in Cuf-TMB disappeared. Both peak positions represent the unsubstituted phenyl C-H out-of-plane bending vibration, which further proves that the C-H on the benzene ring is changed. This provides favorable evidence for us to deduce the structure model of Cuf-TMB.

Table S2. Analysis of FTIR spectra of Cuf-TMB NPs.

**Wavenumbers (cm^{-1})**	**Functional groups**	**Ref.**
3384, 2922	O-H stretching	[14]
3248, 2737	N-H vibration	[15]
1632	-C(=O)H	[16]
1473	$\delta_a(CH_3)$	[17]
1439, 1092	ν_s(C-N)	[18, 19]
1213	δ(C-H)	[12]
862	C–H deformation of phenyl ring	[20]

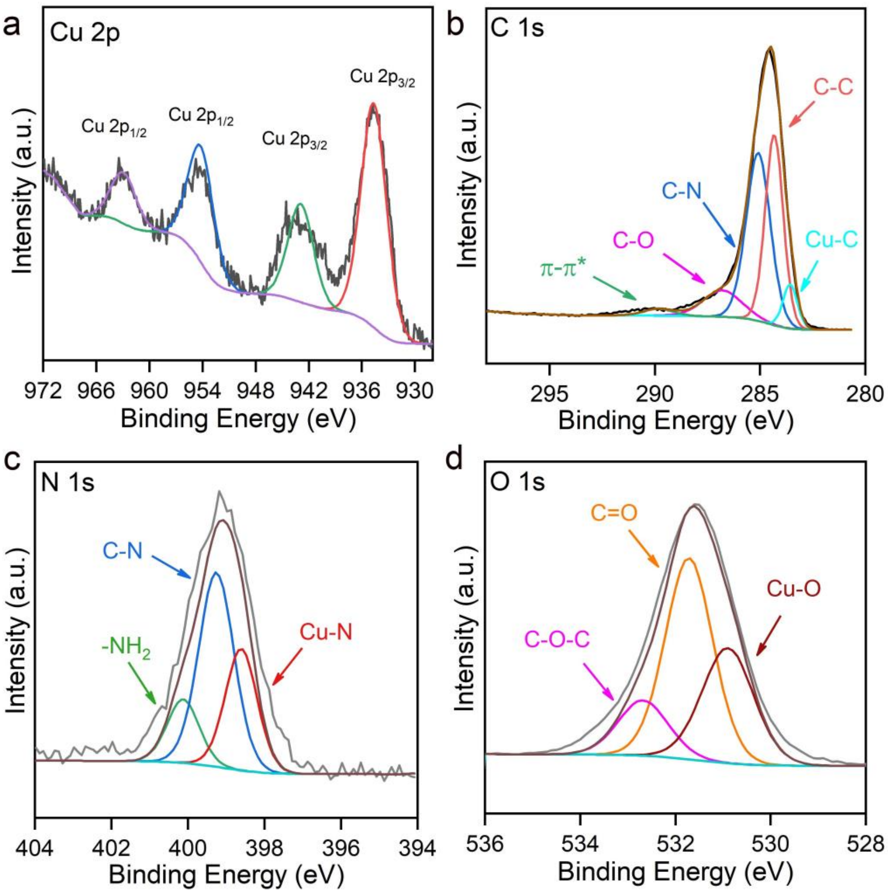

Figure S9. Fine XPS spectra of Cuf-TMB NPs: (a) Cu 2p; (b) C 1s; (c) N 1s; (d) O 1s. XPS spectra were carried out to investigate the binding states of C, N, Cu and O on Cuf-TMB NPs. The Cu 2p XPS spectra of Cuf-TMB NPs showed 933.6 eV and 953.3 eV, which are typical for Cu^{2+}. The peaks at 944.1 eV and 963.0 eV belong to the satellite peaks of Cu(II), further confirming the presence of Cu for Cuf-TMB, to further determine the valence state of Cu. The C 1s spectrum was well-fitted with three peaks at 290.1 eV (benzene ring), 287.2 eV (C-O), 285.8 (C-N), 284.8 eV(C-C), and 283.6 (Cu-C). [21] For the N 1s peak of Cuf-TMB, three peaks corresponding to 399.6 eV (Cu-N), 400.8 eV(C-N), and 400.1 eV(-NH_2) were observed, respectively.[22] The O 1s spectrum was well-fitted with three peaks at 530.9 eV (Cu-O), 531.7 eV (C=O), and 532.6 eV (C-O-C). The results confirmed that C and N species were coated around Cu(II).

Table S3. Elemental analysis of Cuf-TMB NPs.

**Elemental composition**	**Percentage**
Cu	8.59%
N	6.41%
O	21.82%
C	57.16%
H	6.03%

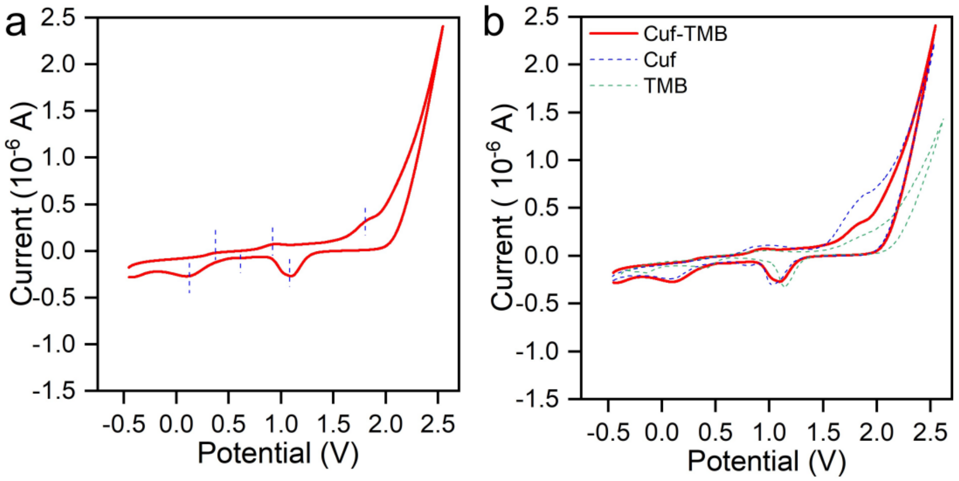

Figure S10. (a) Cyclic voltammetry (CV) of Cuf-TMB NPs. (b) CV of Cuf-TMB NPs, Cuf, and TMB. To evaluate the chemical environment of the Cu element, *in situ* cyclic voltammetry (CV) measurements of Cuf-TMB suspensions were performed. The CV measurements further confirmed that the chemical environment of Cu was Cu(II) species, and ·OH radicals can be captured in the suspension.

Table S4. Analysis of different peaks from the CV curves of Cuf-TMB NPs.

Redox Potential (V)	Attributed Peaks	Ref.
0.37	O atom in organic ligands	[23]
0.91	·OH	[24]
1.79	$HCOO^-$	[25, 26]
0.12	Cu(I) to Cu(0)	[23]
0.61	·OH	[25]
1.07	Cu(II) to Cu(I)	[23, 27]

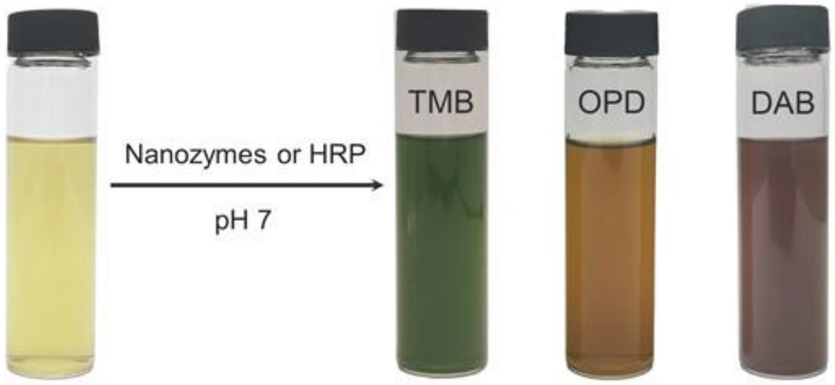

Figure S11. To characterize the peroxidase-like activity of Cuf-TMB, we conducted experiments replacing TMB with other peroxidase substrates, namely di-azo-aminobenzene (DAB) and o-phenylenediamine (OPD). The results demonstrated that Cuf-TMB exhibited catalytic activity not only towards TMB, resulting in a deep blue color, but also towards DAB and OPD, leading to a brown color and orange color. These findings clearly indicate that Cuf-TMB possesses peroxidase-like activity towards commonly used peroxidase substrates. Under the reaction conditions of room temperature and pH 7, the concentrations of TMB, DAB, and OPD were uniformly set at 2 mM for the color development reactions depicted in the images. Each reaction was allowed to proceed for a standardized duration of 5 minutes.

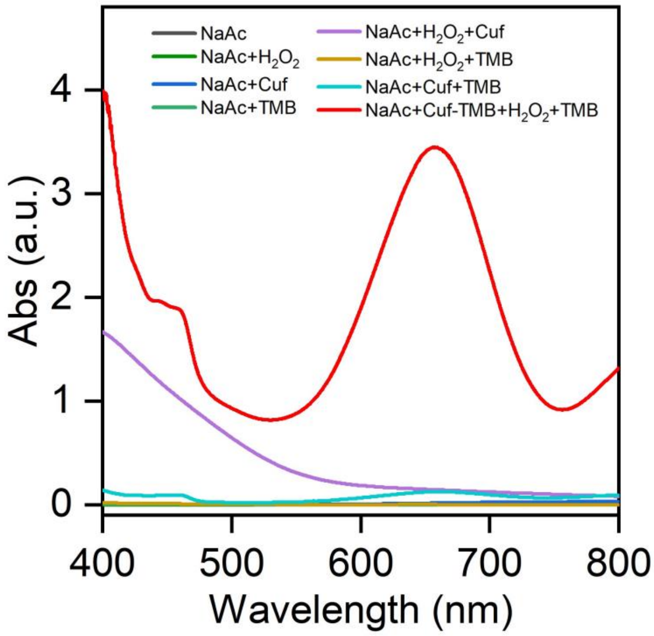

Figure S12 The UV-Vis absorption spectra of Cuf-TMB NPs and other controls. Cuf-TMB NPs show better POD-like catalytic properties. The POD-like activity of Cuf-TMB was systematically investigated by monitoring the UV-vis absorbance at 652 nm for the oxidation form of 3,3',5,5'tetramethylbenzidine (TMB) substrates (oxTMB) in the presence of H_2O_2 (red curve). In contrast, the negligible absorbance of the solution containing only Cuf-TMB and TMB excludes the oxidase (OXD)-like activity of Cuf-TMB (light green). The catalytic activity of freestanding controls was also compared and neither POD-like nor OXD-like activity was found, suggesting the negligible activity of controls.

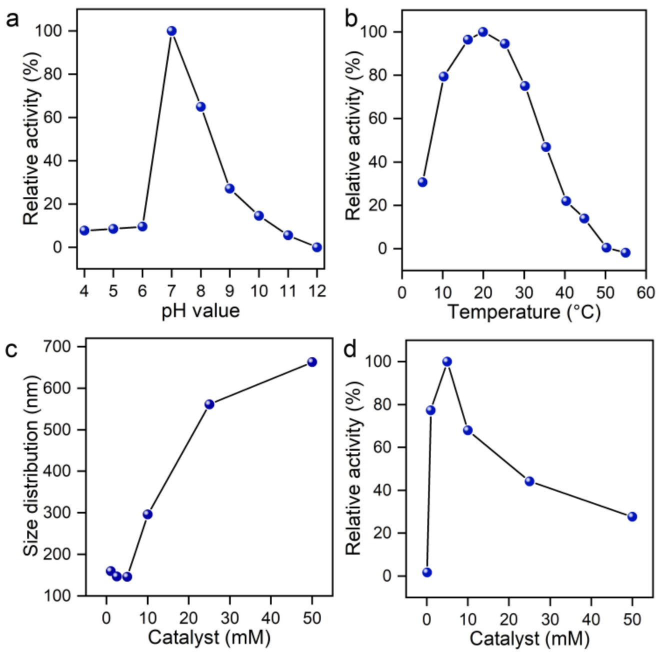

Figure S13. (a) Effect of pH value on the POD-like activity of Cuf-TMB NPs. The POD-like activity of Cuf-TMB was evaluated at different pHs, which shows good performance at pH 7. (b) Effect of temperature on the POD-like activity of Cuf-TMB NPs. The absorbance at 652 nm shows a peak at 20 ℃ within a temperature range of 5 ℃ to 55 ℃. Thus, 20 ℃ is selected as the optimal temperature during the experiment. The relative activity on the Y-axis is defined as the strongest absorption measured at 25 ℃ or pH=7 as 100%, and the absorbance for other conditions is defined as the relative activity under that condition. (c) Effect of Cuf concentration on the POD-like activity of Cuf-TMB NPs. The size distribution of Cuf-TMB NPs derived from different Cuf concentrations. The particle size distribution was obtained from the DLS instrumentation so the reactor rated particle size is an average particle size rather than an individual size. (d) The effects of Cuf concentration on the POD-like activity of Cuf-TMB NPs. The concentration of Cuf-TMB also has an influence on the size distribution and catalytic reaction. At a concentration of 5 mM Cuf, the smallest nanoparticle size was observed, which corresponded to the highest catalytic activity. This effect can be attributed to the larger surface-to-volume ratio of smaller nanoparticles, enabling enhanced substrate interaction.

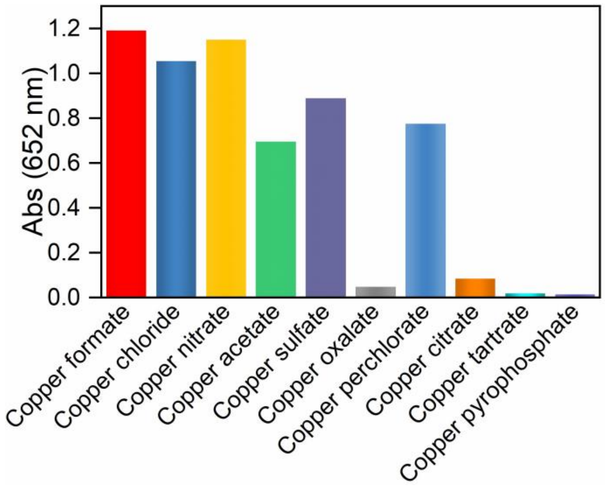

Figure S14. Effect of anion on the POD-like activity of CuX-TMB NPs. To investigate the effect of anions, different copper salt was used to synthesize the CuX-TMB NPs. Furthermore, their POD-like activity was recorded by UV-vis at 652 nm. Among them, Cuf-TMB demonstrated the best performance. Comparison of the POD-like enzyme activities of CuX-TMB revealed that Cuf-TMB demonstrated the best performance, which may be related to the unique structure of Cuf.

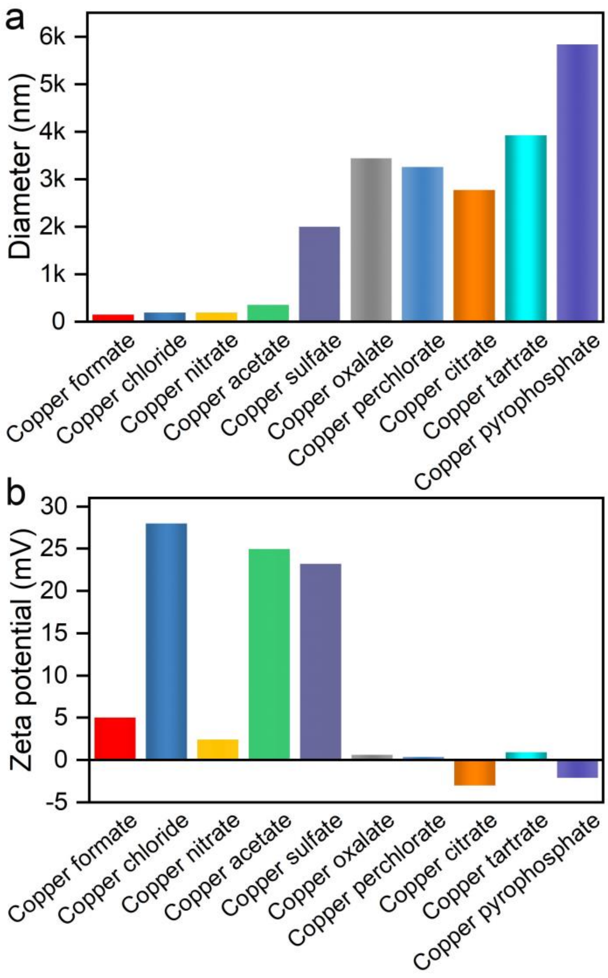

Figure S15. The particle size (a) and zeta potential (b) of the CuX-TMB NPs. To further study the morphology of CuX-TMB NPs, the size distribution and Zeta potential were measured. Among of CuX-TMB, Cuf-TMB showed the smaller size and the proper zeta potential (4.98 mV). Zeta potential data is associated with colloidal stability. Zeta potential provides information on the electrostatic repulsive Forces. However, zeta potential does not provide any insight on the attractive van der Waals forces. Therefore, it is not uncommon to come across stable colloids with low zeta potential. Mild electrostatic repulsion reflected by low zeta potential may be enough to ensure colloid stability. DLVO theory could explain the stability of Cuf-TMB colloidal system, in which 4.98 mV of zeta potential, mild electrostatic, may be enough to ensure colloid stability.[27] The zeta potential 4.98 mV at pH 7 can also be stably present explained according to the above theory, in the Cuf-TMB colloidal system.

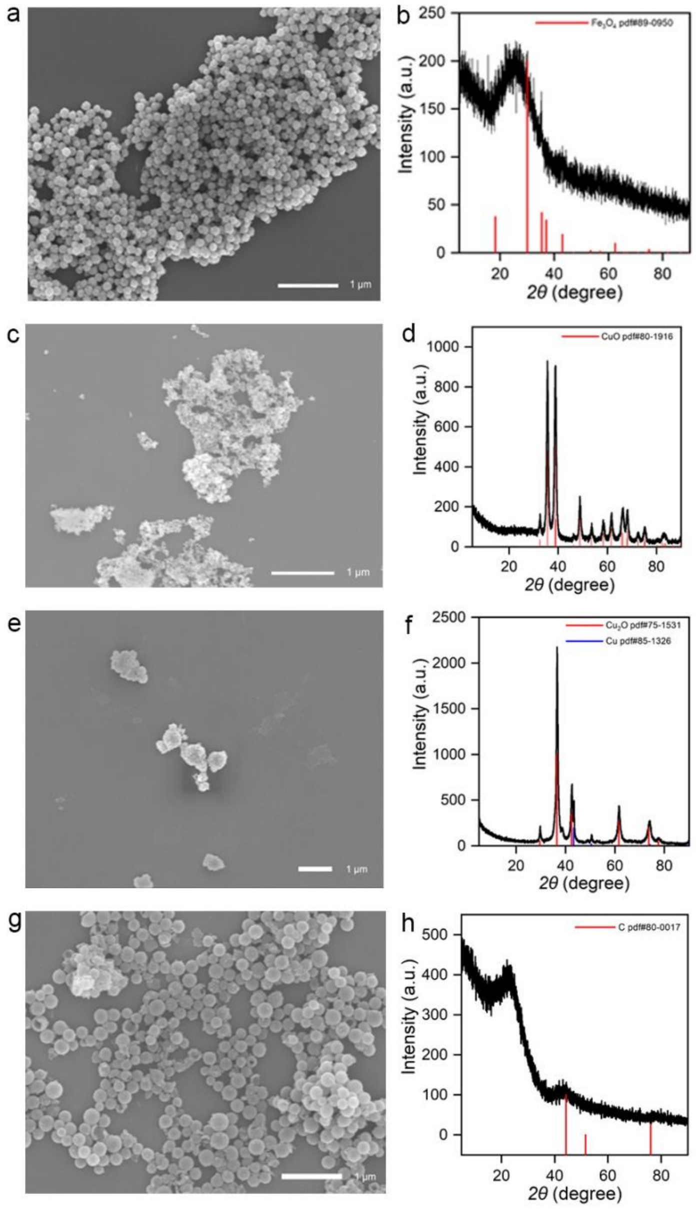

Figure S16. SEM images (a, c, e, g)**,** and XRD pattern (b, d, f, h) of commercially purchased Fe_3O_4, CuO, Cu_2O and C NPs. In order to determine whether the morphology and structure of commercially purchased conventional nanozymes meet the requirements, we did SEM and XRD characterization of Fe_3O_4, CuO, Cu_2O and C NPs. It was confirmed that the commercially purchased conventional nanozymes meet the requirements. The peroxidase activity can be further compared.

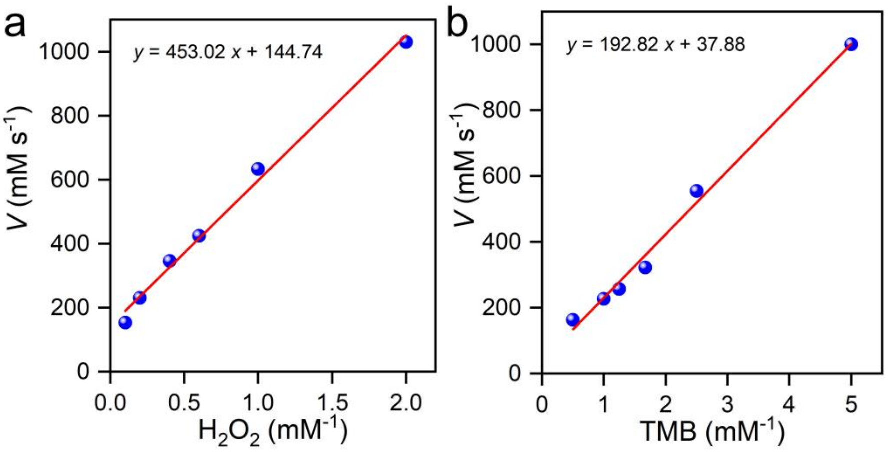

Figure S17. Double-reciprocal plots of activity of horseradish peroxidase (HRP) at a fixed concentration of one substrate *versus* varying concentration of the second substrate for (a) H_2O_2 and (b) TMB. To investigate the kinetic process of HRP, we measured the catalytic process of HRP and made a double inverse plot for the comparison of the catalytic performance.

Table S5. . The Michaelis-Menten constant of different nanozymes, including C, Fe_3O_4, CuO, and Cu_2O NPs under pH 7. Michaelis-Menten constants for the response of Cuf-TMB, CuO, Cu_2O, Au, and HRP nanozymes to the H_2O_2 substrate were calculated. [E/Catalyst] is the molar concentration of the catalyst active site, K_m is the Michaelis constant, V_{max} is the maximum reaction rate, and K_{cat} is the catalytic constant, where $k_{cat} = V_{max}$/[E/Catalyst] and the K_{cat}/K_m value indicates the catalytic efficiency of the enzyme or nanozyme (n=3 independent measurements).

**Catalyst**	**Substrate**	**E/Catalyst**	**K_m (mM)**	**V_{max} (M s^{-1})**	**K_{cat} (s^{-1})**
Cuf-TMB	H_2O_2	1.55×10^{-9}	1.02×10^{-5}	4.8×10^{-11}	3.09×10^{-2}
CuO	H_2O_2	1.07×10^{-3}	0.071	5.46×10^{-4}	0.51
Cu_2O	H_2O_2	5.35×10^{-4}	0.166	3.19×10^{-3}	5.96
Au	H_2O_2	0.099	33	6.1×10^{-8}	6.1×10^{-7}
HRP	H_2O_2	6.80×10^{-8}	0.78	4.39×10^{-4}	5.35×10^{4}

Table S6. The Michaelis-Menten constant of different nanozymes, including C, Fe_3O_4, CuO, and Cu_2O NPs under pH 4.

Materials	Substrate	E/Catalyst	K_m(mM)	V_{max}(M s^{-1})	K_{cat}(s^{-1})
C	H_2O_2	5.35×10^{-4}	3.71×10^{-2}	7.45×10^{-5}	0.139
	TMB		0.02	2.89×10^{-4}	0.54
Fe_3O_4	H_2O_2	3.56×10^{-4}	4.32×10^{-3}	5.5×10^{-3}	1.02
	TMB		2.31×10^{-2}	2.75×10^{-3}	5.13
CuO	H_2O_2	6.99×10^{-6}	3.49×10^{-3}	3.74×10^{-3}	8.86
	TMB		7.70	2.85×10^{-4}	0.53
Cu_2O	H_2O_2	2.67×10^{-4}	0.05	6.55×10^{-4}	24.48
	TMB		0.84×10^{-5}	8.35×10^{-2}	3.08

The calculation values of the Michaelis-Menten constant at pH 4 for peroxidase mimics are relatively well-developed. Samples Fe_3O_4, C, and CuO were purchased from Xianfeng Nano, and Cu_2O was purchased from Sci-Tech.

Table S7. The Michaelis-Menten constant of different nanozymes, including Au, CuO, Cu_2O, and Cuf-TMB NPs at pH 7.

Materials	Substrate	E/Catalyst	K_m(mM)	V_{max}(M s^{-1})	K_{cat}(s^{-1})
Au	TMB	0.099	11.2×10^{-3}	8.30×10^{-8}	8.3×10^{-7}
CuO	TMB	1.07×10^{-3}	0.002	4.72×10^{-4}	0.44
Cu_2O	TMB	5.35×10^{-4}	0.059	1.28×10^{-3}	2.39
Cuf-TMB	TMB	1.55×10^{-9}	2.14×10^{-5}	2.39×10^{-10}	0.15

Michaelis-Menten constant was calculated by catalyst activity. The concentration of H_2O_2 used 10 mM and the TMB concentration varied from 0 to 2 mM.

Table S8. TON and TOF of different POD-like catalysts, including Cuf-TMB, Fe_3O_4, C, CuO, Cu_2O, Au, and HRP.

Catalyst	TON*	TOF (h^{-1})*
Cuf-TMB	8.914	1069.72
Fe_3O_4	0.091	10.96
C	0.023	2.77
CuO	0.143	17.22
Cu_2O	0.224	26.88
Au	0.879	105.52
HRP	3.956	474.72

* TON and TOF were obtained at a reaction time of 30 seconds.

Although HPR has catalyzed in the first 30 seconds of the reaction, the absorbance of 652 nm fluctuated somewhat in the rest 270 seconds. Comparison of TON and TOF of different nanozymes provides a direct comparison of their catalytic conversion efficiencies. We found that Cuf-TMB exhibited the best TON and TOF.

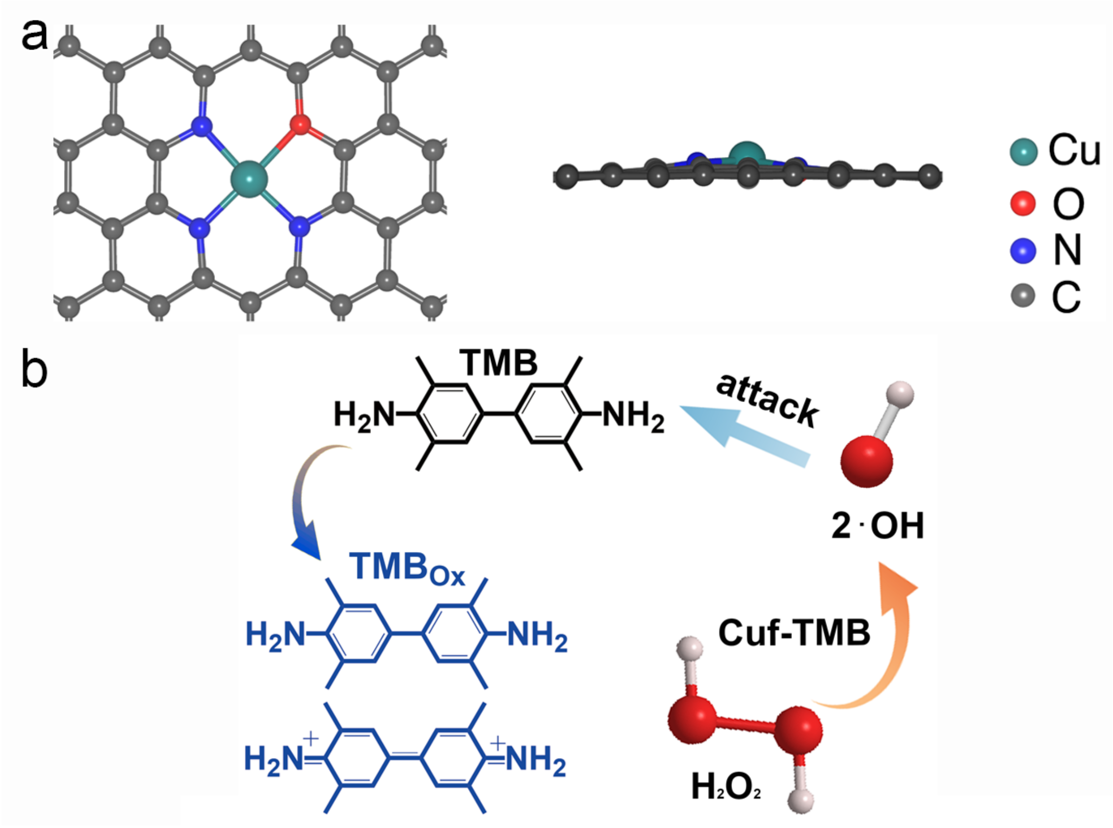

Figure S18. (a) The top view (left) and side view (right) of the model structure of CuN_3O are depicted. (b) Schematic illustration of the origin of Cuf-TMB oxides as peroxidase mimetics is shown. According to the XAFS results, elemental analysis, XPS results, and DFT energy comparisons of Cu-N and Cu-O in Cuf-TMB models, this catalyst model of Cuf-TMB was constructed for the calculations (the top and side view structures are shown).

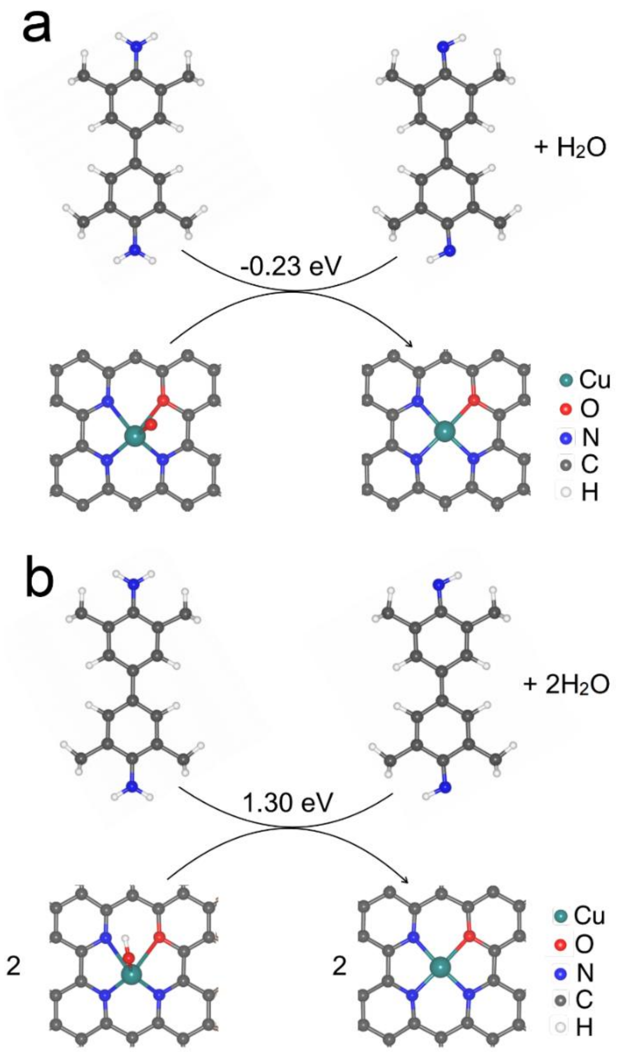

Figure S19. (a) The reaction profile of oxidation of peroxidase substrates by the surface *O species. (b) The reaction profile of oxidation of peroxidase substrates by the surface *OH species. The oxidation of POD substrates by surface *O species requires -0.23 eV (TMB + *O → oxTMB + H_2O(g)), much lower compared to the oxidation by surface *OH species (TMB + 2*OH → oxTMB + 2H_2O(g)). Thereby, the formation of *O intermediate species from *OH species is favourable for the oxidation reaction pathway of POD substrates on the CuN_3O model.

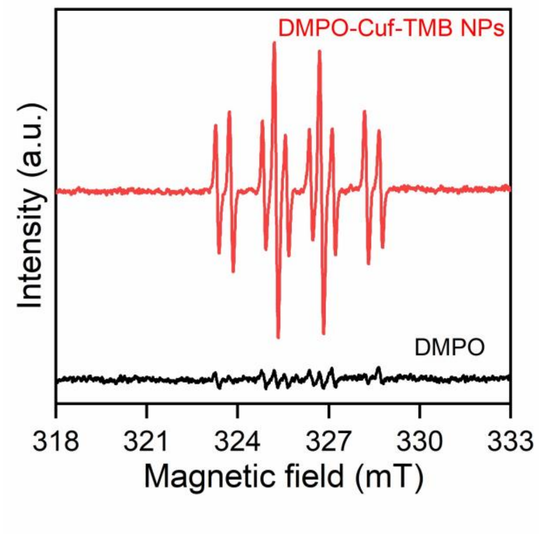

Figure S20. ESR spectra of Cuf-TMB NPs and DMPO. ESR was used to study the mechanism of Cuf-TMB, revealing Cuf-TMB itself contains signals of ·CHO and ·OH radicals. We recorded the ESR spectra of raw materials and products after adding DMPO, a spin-trapping agent of radicals, into the initial reaction mixture. A distinct 1:2:2:1 quartet pattern with a splitting of 1.5 mT of ESR signals was attributed to ·OH, and another 1:1:1:1:1:1 sixtern pattern signals correspond to the signals of the ·CHO.

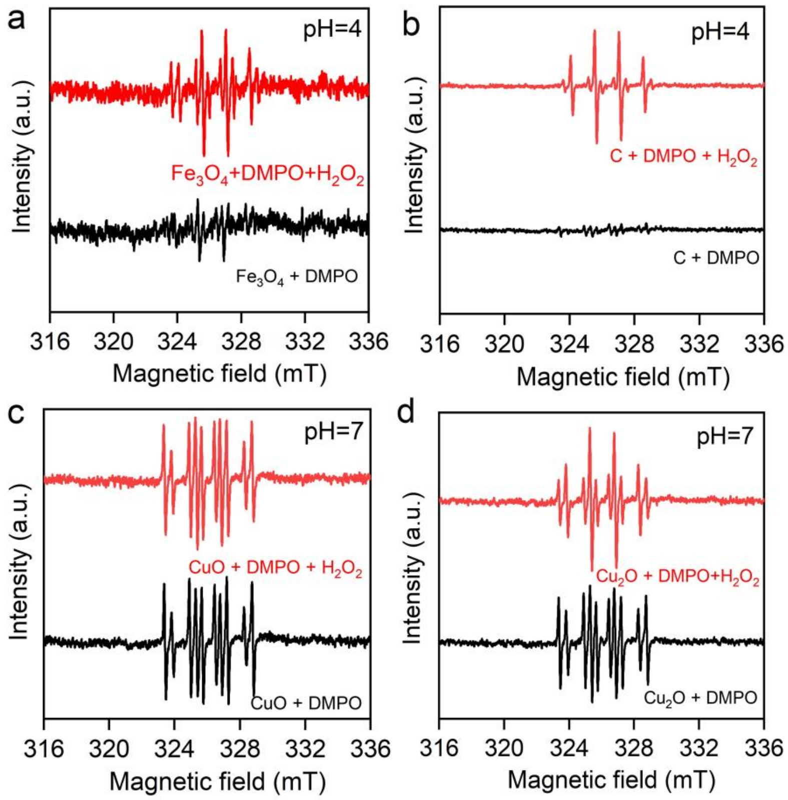

Figure S21. ESR spectra of (a) Fe_3O_4 NPs, (b) C NPs, recorded under the pH 4 conditions, and (c) CuO NPs, (d) Cu_2O NPs, recorded under the pH 7 conditions. We observed the characteristic signals of hydroxyl radicals (1:2:2:1 quartet pattern), upon the addition of 10 mM H_2O_2, 10 mM DMPO and 0.167 mM Fe_3O_4 NPs, C NPs in 10 mM acetate buffer (pH=4), and CuO NPs, Cu_2O NPs in 10 mM acetate buffer (pH=7). ESR was used to identify the POD-like activities of traditional nanozymes. Free radical signals did not catch Fe_3O_4 or carbon nanozymes themselves, but the addition of H_2O_2 revealed ·OH and O_2^{-}· ESR spectra. In the CuO system, DMPO-OH signals can be detected even without any H_2O_2, because of the presence of other origins of this adduct in addition to the genuine spin trapping of ·OH by DMPO. ·OH in the detected DMPO-OH adduct originates 100% from water in the Cu(II) alone system but the amount of ·OH is over 99.8% from the oxidant while H_2O_2 is added. Similar evidence for the hydroxide adduct signal has been found in the Cu_2O system. In addition, the presence of hydrogen peroxide enhances the DMPO-OH radical signal.

Table S9. Effect of different amine ligands on the POD-like activity of Cuf-amine NPs. *The POD-like activity of Cuf-TMB was defined as 100%, relative activity values mean the relative POD-like activity compared to Cuf-TMB.

No.	Name	The structural formula of the amine ligand	Relative activity
1	3,3',5,5'-Tetramethylbenzidine	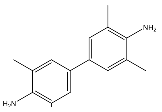	100%
2	3,5 dimethylpiperidine	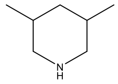	42.12%
3	2,4,6 dimethylaniline	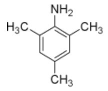	54.12%
4	3,5 dimethylaniline	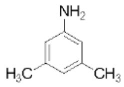	42.11%
5	2,3 dimethylaniline	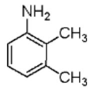	44.24%
6	2,5 dimethylaniline	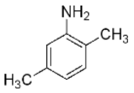	40.23%
7	2,4 dimethylaniline	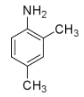	48%
8	3,4 dimethylaniline	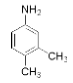	60.24%
9	4,4 dimethylaniline	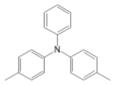	64%
10	Triphenylamine	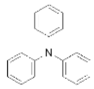	79.06%
11	pyrrole	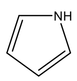	38.59%
12	Laury amine	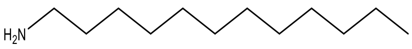	21.65%
13	Hexadecylamine	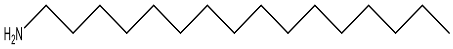	53.18%
14	Octylamine	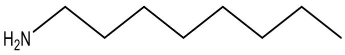	23.53%

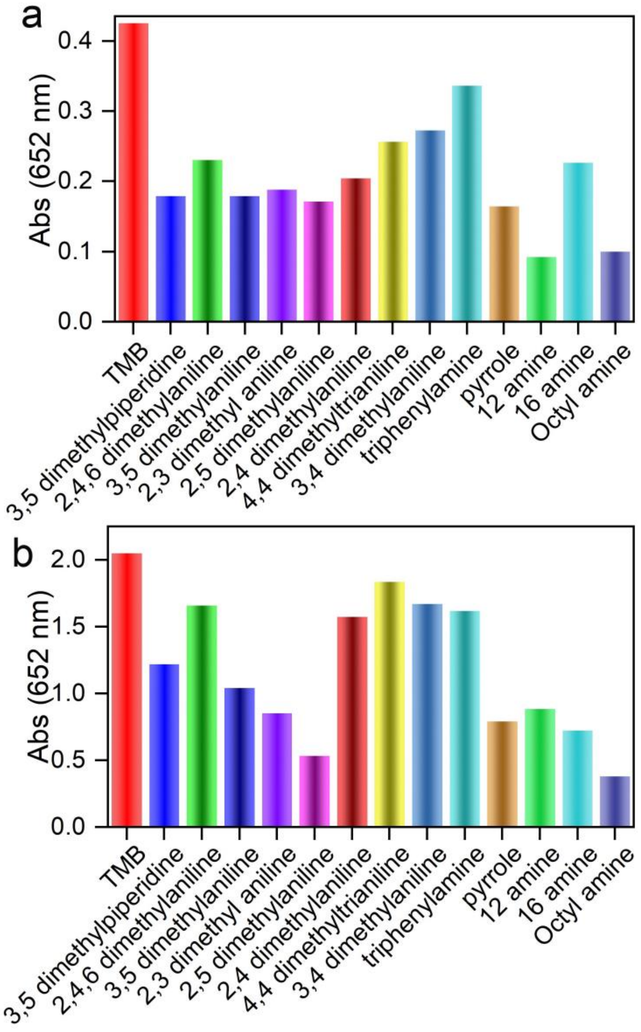

Figure S22. Effect of different amine ligands on the POD-like activity of Cuf-amine NPs. The absorbance at 652 nm was measured to compare the nanozyme properties of the Cuf-amine NPs with different substrates: (a) OPD and (b) TMB. TMB derivatives with methyl and phenyl substituents were designed to gain insight into the structural and coordinating effects of Cuf-TMB on radical stabilization and catalytic activity. Cuf-amine NPs with the TMB ligand show better POD-like activity.

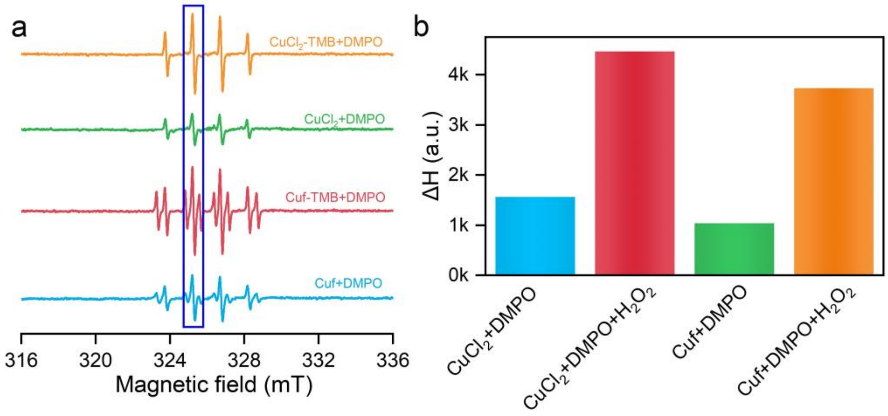

Figure S23. Comparison of the ESR signal for synthesized CuX-TMB NPs from copper chloride and copper formate. (a) ESR spectra. (b) Intensity comparison of ESR signal. Compared with the $CuCl_2$-TMB NPs, the ESR spectra of Cuf-TMB shows the ·CHO and ·OH radicals, while $CuCl_2$-TMB only shows the signal of ·OH. It demonstrated that format ions played an important role in stabilizing radicals of the Cuf-TMB system. The influence of formate in stabilizing radicals of Cuf-TMB system was studied by comparing the ESR spectra of Cuf-TMB with $CuCl_2$-TMB and other traditional nanozymes. It was demonstrated that no ·CHO species can be captured in $CuCl_2$-TMB, indicating the contribution of formate in transforming and stabilising radicals. The above results suggest that the appropriate anion structure and steric hindrance effect can effectively promote the controlled release of free radicals.

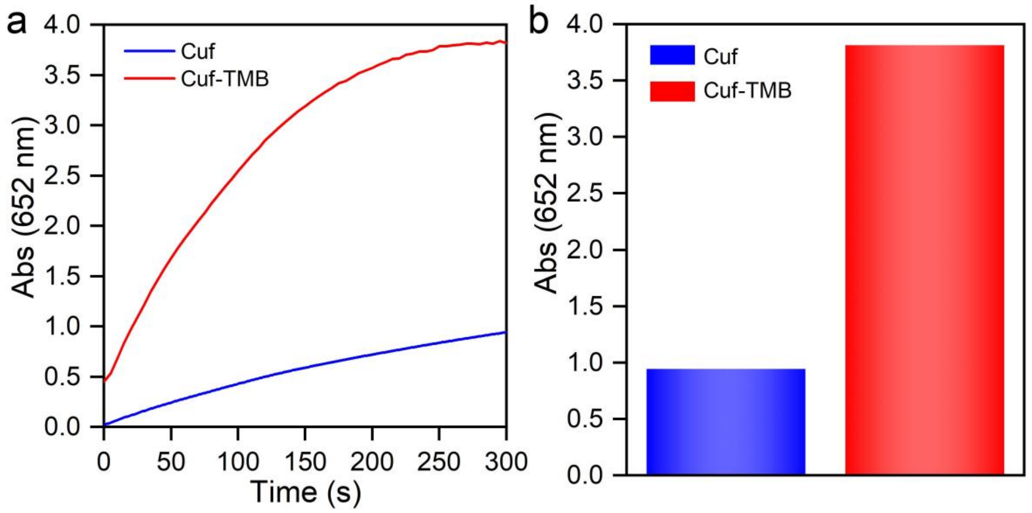

Figure S24. (a) Reaction-time curves of TMB colorimetric reactions catalyzed by Cuf-TMB NPs, and Cuf. (b) Comparison of the specific activities of Cuf-TMB, and Cuf. To claim that Cuf-TMB is special, the POD-like activity of Cuf alone and Cuf-TMB was compared. It was demonstrated that the POD-like activity of Cuf-TMB NPs is 4.05 times to that of Cuf.

Table S10. Effect of different amino acid ligands on the POD-like activity of Cuf-amine NPs. *The POD-like activity of Cuf-TMB was defined as 100%, relative activity values mean the relative POD-like activity compared to Cuf-TMB.

**No.**	**Name**	**The structural formula of the amino acid ligand**	**Relative activity**
1	Lysine	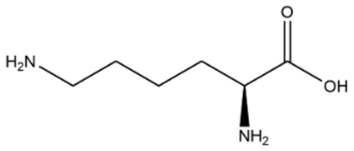	52.0%
2	Histidine	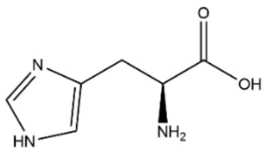	42.9%
3	Alanine	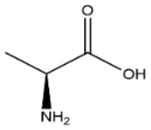	28.9%
4	Glutamic acid	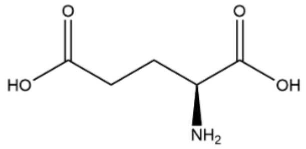	21.3%
5	Cysteine	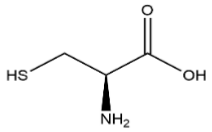	19.4%
6	Tryptophan	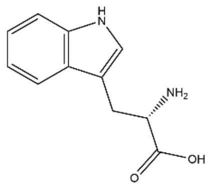	17.4%

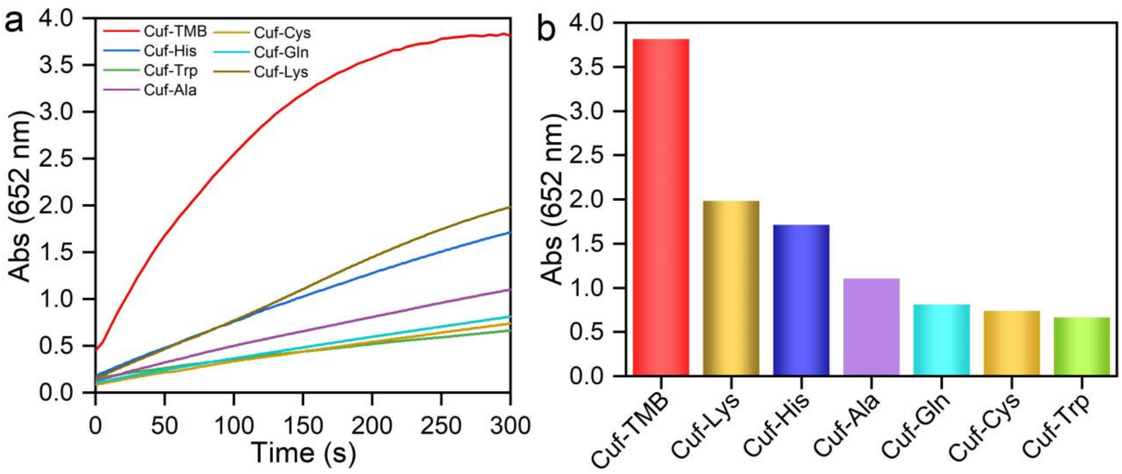

Figure S25. Effect of different amino acid ligands on the POD-like activity of Cuf-amine NPs. (a) Reaction-time curves of TMB colorimetric reactions catalyzed by Cuf-TMB NPs and Cuf-amino acid NPs. (b) Comparison of the specific activities of Cuf-TMB and Cuf-amino acid NPs. To claim that Cuf-TMB is special, the POD-like activity of Cuf-amino acid and Cuf-TMB was compared. It was demonstrated that the POD-like activity of Cuf-TMB NPs is 1.92, 2.23, 3.46, 4.69, 5.16, 5.75 times to that of Cuf-Lys, Cuf-His, Cuf-Ala, Cuf-Gln, Cuf-Cys, Cuf-Trp.

Table S11. Effect of different nucleotide ligands on the POD-like activity of Cuf-amine NPs. *The POD-like activity of Cuf-TMB was defined as 100%, relative activity values mean the relative POD-like activity compared to Cuf-TMB.

No.	Name	The structural formula of the nucleotide ligand	Relative activity
1	Guanine	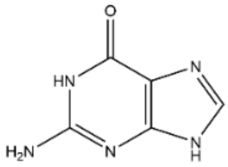	43.1%
2	Thymine	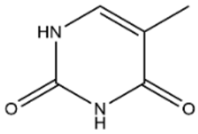	30.1%
3	Cytosine	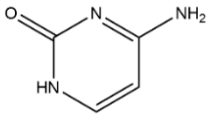	31.0%
4	Adenine	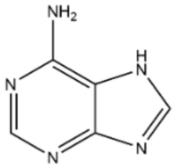	6.5%

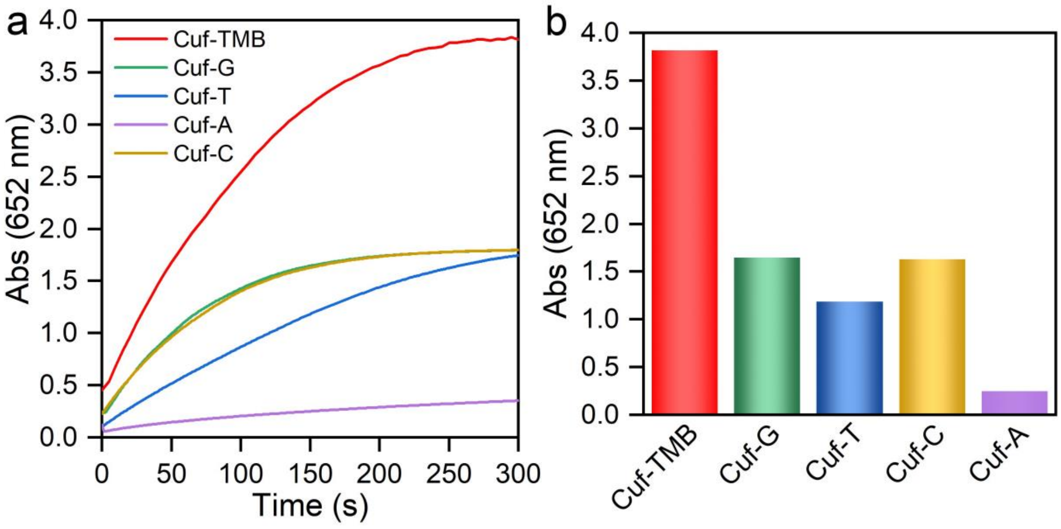

Figure S26. Effect of different nucleotide ligands on the POD-like activity of Cuf-amine NPs. (a) Reaction-time curves of TMB colorimetric reactions catalyzed by Cuf-TMB and Cuf-nucleotide NPs. (b) Comparison of the specific activities of Cuf-TMB and Cuf-nucleotide NPs. To claim that Cuf-TMB is special, the POD-like activity of Cuf-nucleotide and Cuf-TMB was compared. It was demonstrated that the POD-like activity of Cuf-TMB NPs is 2.32, 3.22, 3.27, and 15.26 times to that of Cuf-G, Cuf-T, Cuf-C, Cuf-A.

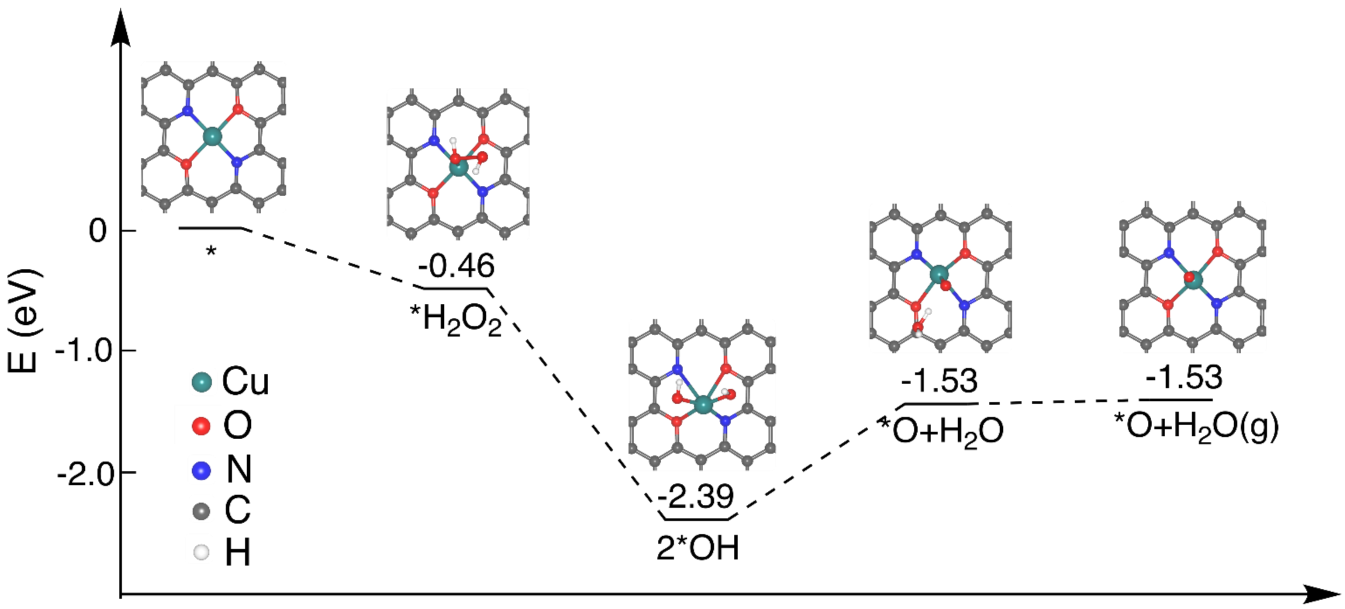

Figure S27. The energy panel of the reaction pathway on the CuN_2O_2 model. The energy profile diagram shows the most favourable path of H_2O_2 dissociation into surface *O species in neutral conditions. DFT calculations with a structure of Cu coordinated with two nitrogen and two oxygen atoms (CuN_2O_2) as a model for other Cu-amine complexes revealed an uphill reaction energy (up to 0.86 eV) for the H transfer process between the two *OH species. This reaction energy is higher than the overall energy barrier in CuN_3O, indicating the superior POD-like activity of Cuf-TMB NPs.

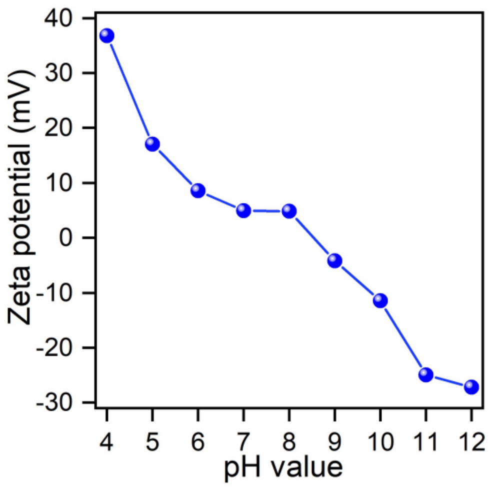

Figure S28. Zeta potential values of the Cuf-TMB NPs under different pH conditions. To further investigate the relationship between the colloidal stability of the Cuf-TMB system and its stable presence of free radicals, we determined its zeta potential at different pH. As per the most widely accepted DLVO (named after inventors Derjaguin, Landau, Verwey and Overbeek) theory, colloid stability depends on the sum of van der Waals attractive forces and electrostatic repulsive forces due to the electric double layer (EDL). Zeta potential provides information on the electrostatic repulsive Forces. However, zeta potential does not provide any insight on the attractive van der Waals forces. Therefore, it is not uncommon to come across stable colloids with low zeta potential. Mild electrostatic repulsion reflected by low zeta potential may be enough to ensure colloid stability. DLVO theory could explain the stability of Cuf-TMB colloidal system, in which 4.98 mV of zeta potential, mild electrostatic, may be enough to ensure colloid stability. The zeta potential of 4.98 mV at pH 7 can also be stably present explained according to the above theory, in the Cuf-TMB colloidal system. Cuf-TMB colloidal system was stably present, and the ESR experiment also proved the radical could exist for 52 days. In summary, 4.98 mV of the zeta potential was more favorable for the stability of radicals

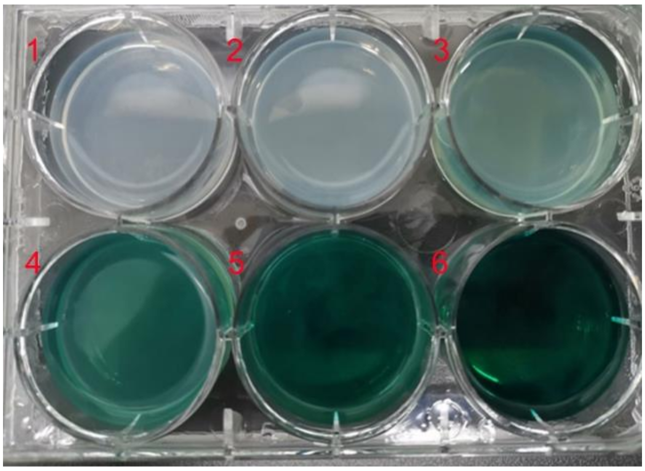

Figure S29. Optical photograph of the POD-like activity at 30 min of Cuf-TMB NPs loaded agarose hydrogels of different volume ratios. The volume ratios of Cuf-TMB NPs suspension (5 mM Cuf-derived) to agarose (1 wt%) were 1:9, 2:8, 4:6, 6:4, 8:2, and 9:1, corresponding to NO.1-6. The volume ratio of 8:2 is the optimized condition. To enable efficient and convenient biochemical monitoring of cholesterol, ALP, and glucose in the clinical setting, we immobilized Cuf-TMB colloidal particles onto agar.

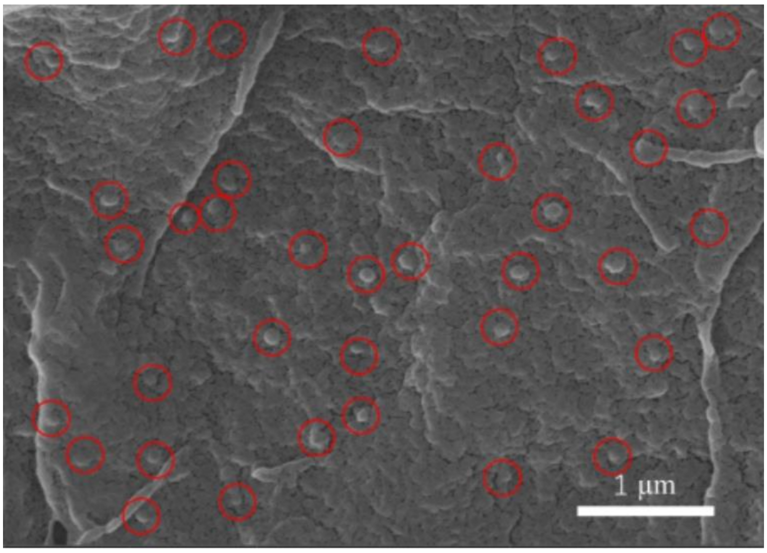

Figure S30. SEM image of Cuf-TMB NPs loaded agarose hydrogels. The Cuf-TMB NPs were marked in the red circle. The SEM images show that Cuf-TMB can be distributed more uniformly on the hydrogel surface.

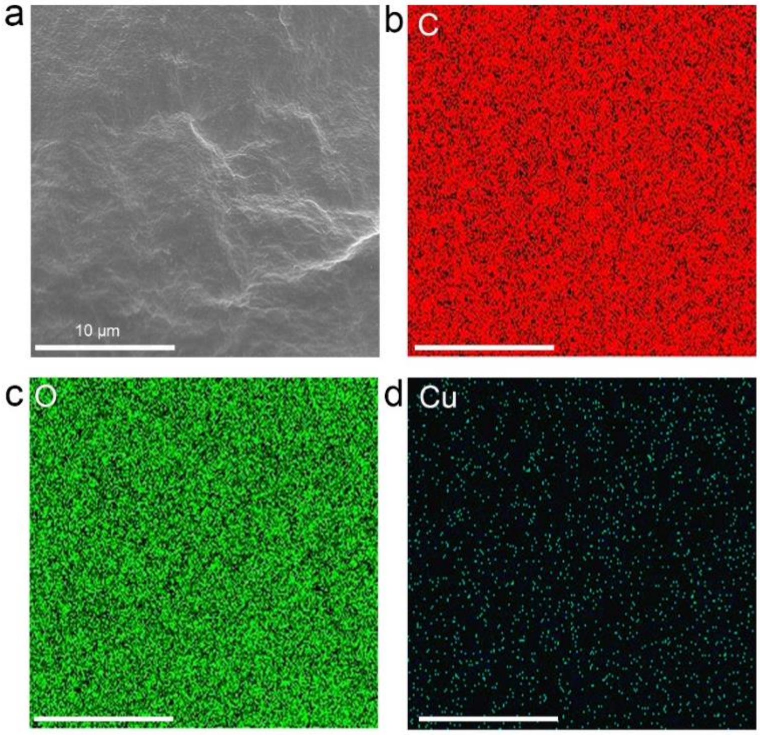

Figure S31. (a) SEM and corresponding EDS mapping of (a) Cuf-TMB NPs. Scale bar, 10 μm. EDS mapping analysis of Cuf-TMB NPs. Color legend: (b) red, C; (c) green, O; (d) blue, Cu. Cuf-TMB was immobilized into a hydrogel and the presence of Cu was still observed by EDS. And the properties of Cuf-TMB-gel were maintained in the subsequent experiments.

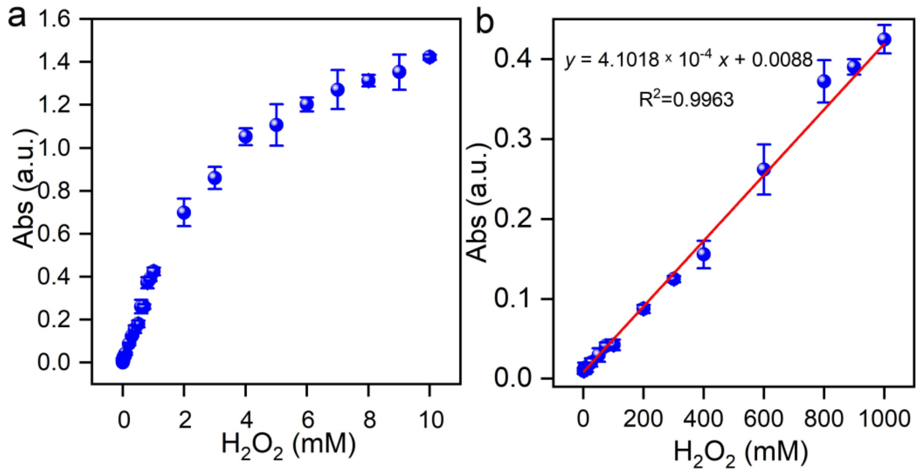

Figure S32. Application of Cuf-TMB NPs for H_2O_2 detection. (a) Response curve of Cuf-TMB NPs for H_2O_2 concentration (0-10 mM). (b) Linear calibration plot of H_2O_2 (0-1000 μM). Taking advantage of the remarkable POD-like activity, the colorimetric detection of H_2O_2 has been realized. It is critical to have effective performance in the detection of H_2O_2, which is a vital ingredient for the detection of cholesterol and glucose, before it can be utilized for clinical biochemical index testing.

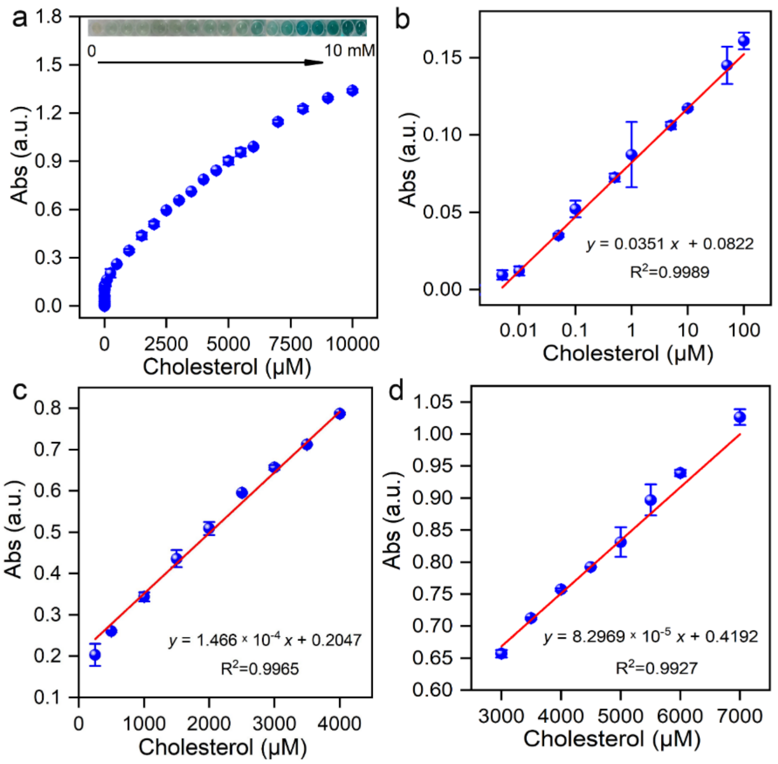

Figure S33. (a) Response curve of cholesterol (0-10 mM) with Cuf-TMB NPs-based biosensor. Inset: corresponding optical photographs. (b-d) Linear calibration plot for cholesterol of different concentrations. Detection of cholesterol by a relatively wide detection range and the presence of 3 linear ranges within that range is the basis for clinical testing.

Table S12. Performance of cholesterol detection by different biosensors.

Materials	LOD	Methods	Ref
Zn-Mo single atom	0.76 μM	colorimetric	[28]
Zn-Mo single atom	7.44 μM	colorimetric	[28]
PPy NPs	3.5 μM	colorimetric	[29]
CuO NPs	0.43 μM	electrochemical	[30]
Au NPs modified cholesterol oxidase	34.6 μM	electrochemical	[31]
MWCNT@MIP-CCEs	1 nM	electrochemical	[32]
$CoCl_2$	2 μM	electrochemical	[33]
PANI/MWCNTs/Starch nanocomposite	10 μM	electrochemical	[34]
Cu_2S NRS/Cu rod	0.1 μM	electrochemical	[35]
NiO/CVD-grown graphene	0.13 μM	electrochemical	[36]
Cu_2O NPs/TNTs	0.05 μM	electrochemical	[37]
Calix[6]arene functionalized graphene	0.2 μM	electrochemical	[38]
Ru-Pi on PPy/CFP electrode	0.054 nM	electrochemical	[39]
CAT/ChOx/GR-IL/GCE	0.05 μM	electrochemical	[40]
ZnO/ZnS microtubes	0.02 mM	electrochemical	[41]
CPH/PtNPs/enzyme hybrid electrode	0.3 mM	electrochemical	[42]
ZnO hollow nanospheres	0.4 mM	electrochemical	[43]
PTBA/FAD/apo-ChOx electrode	0.22 μM	electrochemical	[44]
Pt/rGO/P3ABA	40.6 μM	electrochemical	[45]

Table S13. Analysis of Cholesterol content in spiked human blood serum samples.

**NO.**	**Normal cholesterol Concentration(mM)**	**Cho-added (50 μM)**	**Recovery (%)**	**Cho-added (500 μM)**	**Recovery (%)**
1	4.88	4.92	96%	5.41	105%
2	3.19	3.23	105%	3.71	103%
3	4.88	4.93	102%	5.36	96%
4	6.14	6.18	89%	6.64	100%
5	6.14	6.19	107%	6.63	98%
6	7.98	8.02	94%	8.51	106%
7	1.55	1.60	107%	2.02	95%
8	3.29	3.34	106%	3.80	102%

To testify the dependability of the standard curves as well as the applicability of Cuf-TMB in real sample detection, the real samples were prepared by diluting the human serum to a low concentration and extra addition of cholesterol to a high concentration, respectively, which exhibited a satisfactory recovery rate.

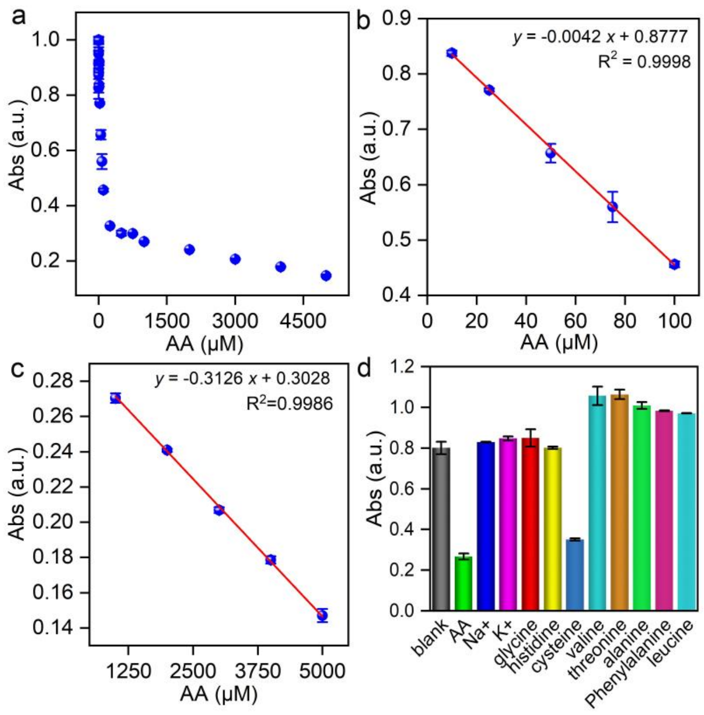

Figure S34. (a) Response curve of AA (0-4.5 mM) with Cuf-TMB NPs-based biosensor. (b-c) Linear calibration plot for AA of different concentrations. (d) Selectivity of Cuf-TMB NPs based biosensor towards AA detection. AA is added into the Cuf-TMB/TMB/H_2O_2 mixture, and the production of oxTMB will be inhibited leading to a rapid decrease in absorbance at 652 nm. Some potential interfering substances (including Na^+, K^+, glycine, histidine, cysteine, valine, threonine, alanine, phenylalanine, and leucine) were employed to investigate the selectivity in AA detection. Most of them posed no interference towards the detection except cysteine.

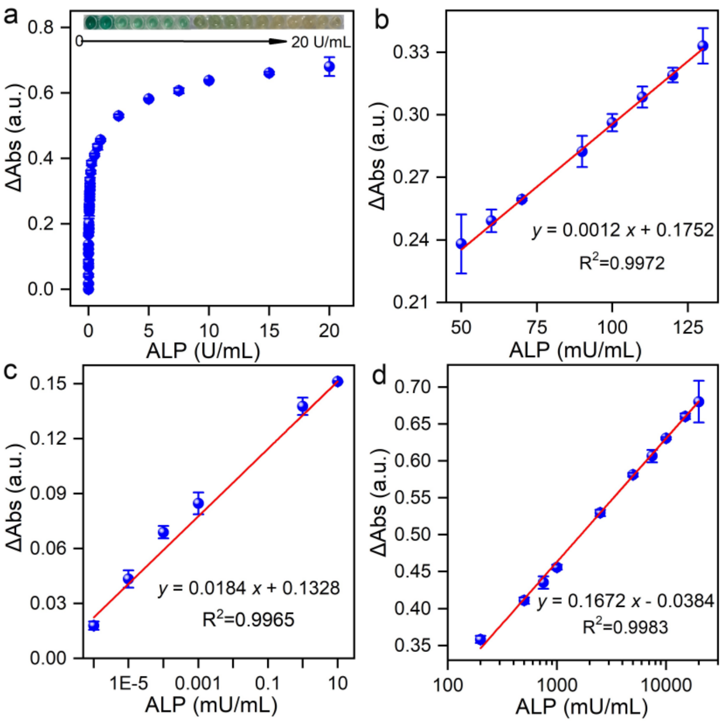

Figure S35. (a) Response curve of ALP (0-20 U/mL) with Cuf-TMB NPs-based biosensor. Inset: corresponding optical photographs. (b-d) Linear calibration plots for ALP of different concentrations. ALP assay is primarily used to diagnose diseases of the hepatobiliary and skeletal systems and is an important indicator of extrahepatic biliary obstruction, intrahepatic space-occupying lesions, and rickets. Therefore, the detection of alkaline phosphatase is of great clinical significance and 3 linear ranges also exist within the range of alkaline phosphatase assay and are the basis of clinical testing.

Table S14. Performance of ALP detection by different biosensors.

**Materials**	**LOD**	**Methods**	**Ref**
Fe/C nanosheets	0.03 U/L	colorimetric	[46]
Fe-N-C single atom	0.05 U/L	colorimetric	[47]
Fe_x-N-C nanozymes	0.03 U/L	colorimetric	[48]
Ir NPs/perovskites	0.39 U/L	colorimetric	[49]
$CsPbBr_3$ QDs@PMMA	4.85 mU/mL	fluorescence	[50]
Carbon dots@Alg/Cu gel	0.87 mU/mL	fluorescence	[51]
Au nanorods	83.2 ng/L	colorimetric	[52]
Pd cube@CeO_2 NPs	0.07 U/L	colorimetric	[53]
Cu NCs@Tb-GMP	0.002 U/mL	fluorescence	[54]
Au NPs	0.39 mU/mL	plasmonic	[55]
Fluorescent DNA Chain	2.6×10^{-6} U/mL	fluorescence	[56]
CeO_2 NPs	0.04 U/L	colorimetric	[57]
Nitrogen-doped graphene QDs	0.07 U/L	fluorescence	[58]
Mn-ZnS QDs@BSA	0.003 U/L	fluorescence	[59]
Nucleic acids@CoOOH nanocomplex	0.027 mU/mL	fluorescence	[60]
MIL-88B-NH_2/Pt NPs	1.89 mU/mL	colorimetric	[61]
CdTe/ZnS QDs	0.65 nM	fluorescence	[62]

Table S15. Analysis of ALP content in spiked human blood serum samples.

NO.	Normal-ALP concentration (mM)	ALP-added (10 mU/mL)	Recovery (%)	ALP-added (25 mU/mL)	Recovery (%)
1	169.00	180.12	110%	193.18	96%
2	155.12	165.40	100%	180.68	100%
3	162.06	170.95	89%	186.79	96%
4	153.06	164.29	102%	176.79	92%
6	44.84	55.12	89%	68.73	95%
7	131.79	142.11	103%	156.79	99%
8	138.73	147.62	85%	163.45	102%

The samples were made by diluting human serum to a low concentration and adding extra ALP to a high concentration, respectively, which demonstrated a satisfactory recovery rate. This was done to demonstrate the dependability of the standard curves as well as the applicability of Cuf-TMB in real sample detection.

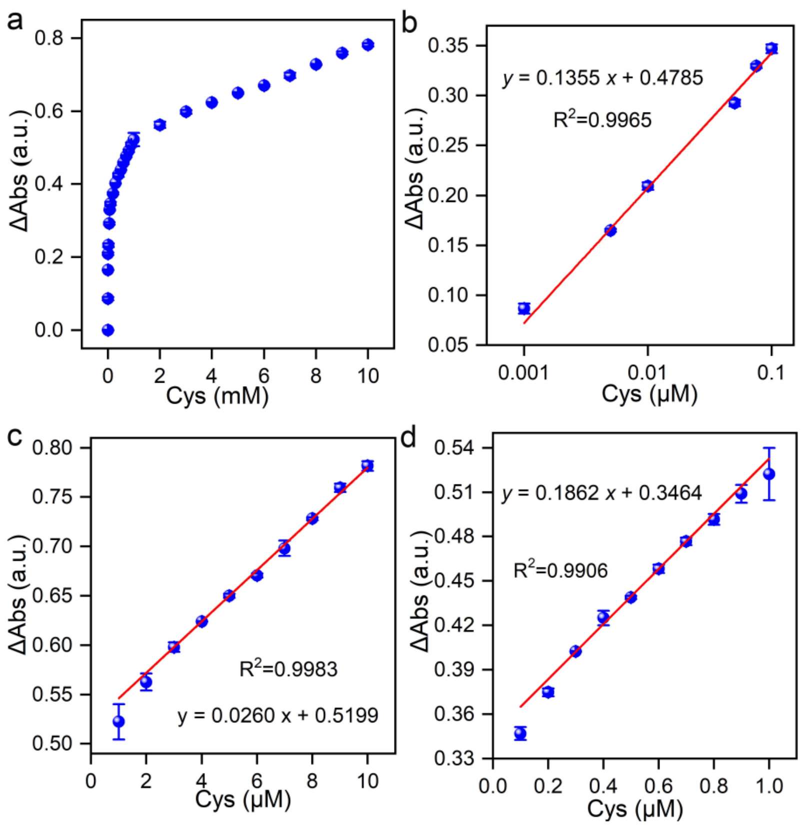

Figure S36. (a) Response curve of Cys (0-10 mM) with Cuf-TMB NPs-based biosensor. (b-d) Linear calibration plots for Cys of different concentrations. Cysteine is a thiol-containing amino acid and can bind with Cu^{2+} via the strong interaction between its thiol groups. Because of the stronger binding affinity between Cu^{2+} and cysteine, the more cysteine is added, the less activity is shown, resulting in a decrease of catalytic ability on the colorimetric reaction of TMB-H_2O_2. The absorption intensity gradually decreases with increasing cysteine.

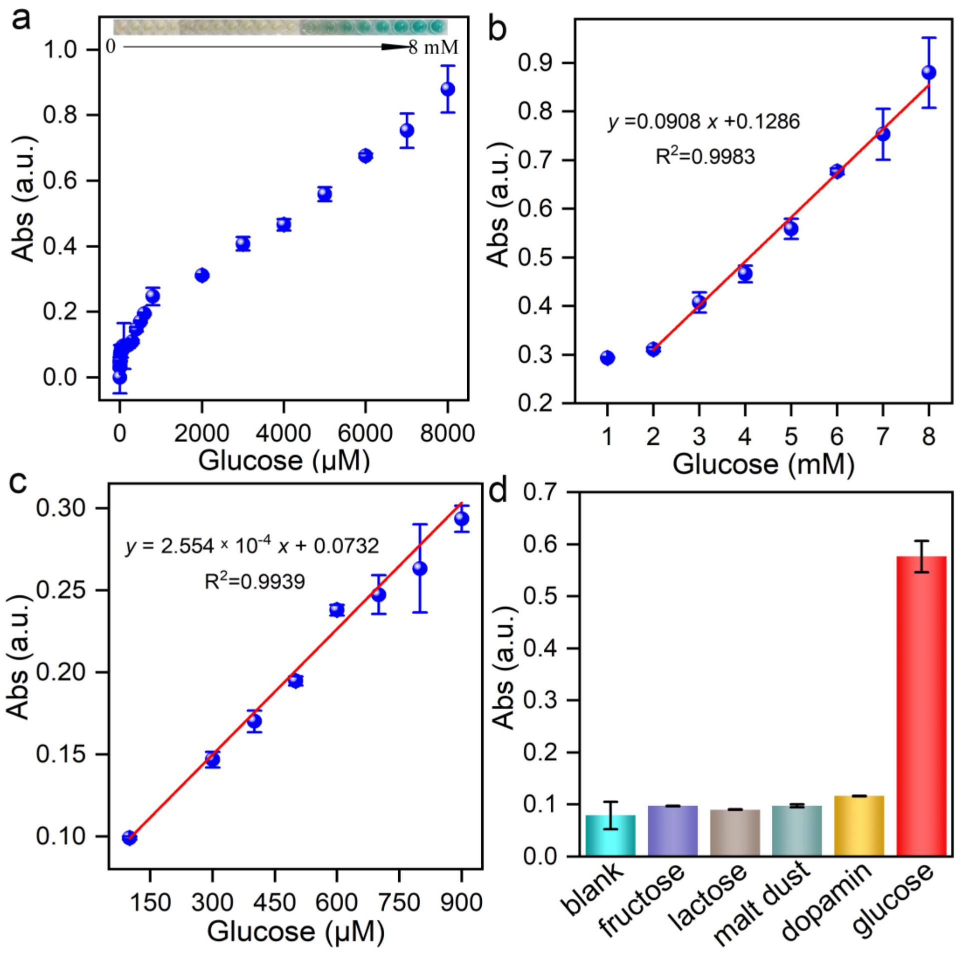

Figure S37. (a) Response curve of glucose (0-8 mM) with Cuf-TMB NPs-based biosensor. Inset: corresponding optical photographs. (b-c) Linear calibration plot for glucose of different concentrations. (d) Selectivity of Cuf-TMB NPs-based biosensor towards glucose detection. Based on the POD-like activity, we also demonstrated the applicability of Cuf-TMB in the detection of glucose.

Table S16. Detection of glucose with different biosensors.

Materials	LOD	Methods	Ref
Zn/Mo DSAC-SMA	15.6 μM	colorimetric	[28]
CuO NPs	0.59 μM	colorimetric	[30]
Cu_xO NPs/PPy NWs/Au	6.2 μM	electrochemical	[63]
Co_3O_4 UHMSA	1.84 μM	electrochemical	[64]
Flower-shaped CuO nanostructures	1.37 μM	electrochemical	[65]

Table S17. Different biochemical indicators in healthy controls and detection limit of this work.

**Biochemical index**	**The normal level in the human body**	**Detection limit of this work**
Glucose	3.9−7.8 mM	90 μM
Cholesterol	3−5.69 mM	0.005 μM
ALP	45−135 mU/mL	10^{-5} mU/mL
AA	60−100 mg	0.001 μM
Cys	5−15 μM	0.001 μM

Supplementary References

1. Zasimov, P. V.; Sanochkina, E. V.; Tyurin, D. A.; Feldman, V. I., An EPR study on the radiolysis of isolated ethanol molecules in solid argon and xenon: matrix control of radiation-induced generation of radicals in cryogenic media. *Phys. Chem. Chem. Phys.* **2023**, *25*, 4624-4634.
2. Christian, P.; Jan, T.; Svend, K. J.; Søren, R. K., Investigation of the Primary Photodynamics of the Aqueous Formate Anion. *J. Phys. Chem.* **2006**, *110*, 3383-3387.
3. Júlio, M.; Rita, T.; Danilo, B. M.; José, P. F. A.; Paolo, D. M.; Nilson, A. A.; Etelvino, J. H. B., Generation of Singlet Oxygen by the Glyoxal−Peroxynitrite System. *J. Am. Chem.Soc.* **2011**, *133*, 20761−20768.
4. Linn, D. E. J.; Gould, E. S.; Electron Transfer. 92. Reductions of Vitamin B12a (Hydroxocobalamin) with Formate and Related Formyl Species. *Inorg. Chem.* **1988**, *27*, 1625-1628.
5. Carter, R. O.; Poindexter, B. D.; Weber, W. H., Vibrational Spectra of Copper Formate Tetrahydrate, Copper Formate Dihydrate and Three Anhydrous Forms of Copper Formate. *Vib. Spectrosc.* **1991,** *2*, 125-134.
6. Canterford, R. P.; Ninio, F., The Raman Spectrum of Copper Formate Tetrahydrate. *J. Phys. C: Solid State. Phys.* **1973,** *6*, 575-582.
7. Heyns, A. M., The Vibrational Spectra of the Copper(II) Formates. *J. Mol. Struct* **1973,** *18*, 471-485.
8. Pohl, M.; Pieck, A.; Hanewinkel, C.; Otto, A., Raman Study of Formic Acid and Surface Formate Adsorbed on Cold-Deposited Copper Films. *J. Raman. Spectrosc.* **1996,** *27*, 805-809.
9. Canterford, R. P.; Ninio, F., The Raman Spectrum of Copper Formate Tetrahydrate. II. *J. Phys. C: Solid. State. Phys.* **1975,** *8*, 385-388.
10. Heyns, A. M.; Range, K. J., The Vibrational Spectra of the Copper (II) Formates Part IV * .The Thermal Behaviour of Cu(HCOO)*4H_2O and Cu(HCOO)*2H_2O. *J. Mol. Struct.* **1987,** *162*, 57-67.
11. Lian, S.; Gao, X.; Song, C.; Li, H.; Chen, A.; Lin, J. Q., The Characteristics of Raman Spectroscopy of Isomer CBD- and THC-Au Nanoparticles Using the Density Functional Theory. *Spectrochim. Acta A Mol. Biomol. Spectrosc.* **2022,** *268*, 120682.
12. Boilet, L.; Buntinx, G.; Lapouge, C.; Lefumeux, C.; Poizat, O., Vibrational and Structural Analysis of the Radical Cation of *N, N, N', N'*-Tetramethylbenzidine Based on Ab-Initio Calculations and Time-Resolved Resonance Raman Spectroscopy. *Phys. Chem. Chem. Phys.* **2003,** *5*, 834-842.
13. Timchenko, P. E.; Timchenko, E. V.; Volova, L. T.; Dolgushkin, D. A.; Boltovskaya, V. V.; Frolov, O. O., Raman Spectroscopy Method for the Evaluation of Bone Bioimplants Made Using the "Lyoplast" Technology from Cadaveric and in Vivo Resected Bone Tissue. *J. Phys,: Conf. Ser.* **2018,** *1038*, 012090.

14. Jiang, X. M.; Li, S. Xiang, G. Q.; Li, Q. H.; Fan, L.; He, L. J.; Gu, K. R., Determination of The Acid Values of Edible Oils Via FT-IR Spectroscopy Based on the OH Stretching Band. *Food. Chem.* **2016,** *212*, 585-589.
15. Geng, X.; Xie, X. N.; Liang, Y. C.; Li, Z. Q.; Yang, K.; Tao, J.; Zhang, H.; Wang, Z., Facile Fabrication of a Novel Copper Nanozyme for Efficient Dye Degradation. *ACS. Omega.* **2021,** *6*, 6284-6291.
16. Yaylayan, V. A.; Ismail, A. A., Investigation of the Enolization and Carbonyl Group Migration in Reducing Sugars by FT-IR Spectroscopy. *Carbohydr. Res.* **1995,** *276*, 253-265.
17. Guichard, V.; Bourkba, A.; Poizat, O., Vibrational Studies of Reactive Intermediates of Aromatic Amines. 2. Free-Radical Cation and Dlcation Resonance Raman Spectroscopy of *N, N, N', N'*-Tetramethylbenzidine and *N, N, N', N'*-Tetraethylbenzidine. *J. Phys. Chem.* **1989,** *93*, 4429-4435.
18. Huang, S.; Yang, E. L.; Yao, J. D.; Chu, X.; Liu, Y.; Zhang, Y.; Xiao, Q., Cobalt Co-Doped Fluorescent Magnetic Carbon Dots as Ratiometric Fluorescent Probes for Cholesterol and Uric Acid in Human Blood Serum. *Acs. Omega.* **2019,** *4*, 9333-9342.
19. Tian, B.; Zhao, L.; Li, R.; Zhai, T.; Zhang, N.; Duan, Z.; Tan, L., Electrochemical Immunoassay of Endothelin-1 Based on a Fenton-Type Reaction Using Cu(II)-Containing Nanocomposites as Nanozymes. *Anal. Chem.* **2020,** *92*, 15916-15926.
20. Li, X.; Zheng, L.; Wang, Y.; Zhang, N.; Lou, Y.; Xiao, T.; Liu, J., A Novel Electrocatalyst with High Sensitivity in Detecting Glutathione Reduced by 2-hydroxypropyl-β-cyclodextrin Enveloped 10-methylphenothiazine. *RSC. Adv.* **2015,** *5*, 71749-71755.
21. Popovici, D.; Czeremuzkin, G.; Meunier, M.; Sacher, E.; Laser-Induced Metal-Organic Chemical Vapor Deposition (MOCVD) of Cu (hfac) (TMVS) on Amorphous Teflon AF1600: An XPS Study of the Interface. *Appl. Surf. Sci.* **1998**, *126*, 198–204.
22. Wang, D.; Ao, C.; Liu, X.; Fang, S.; Lin, Y.; Liu, W.; Zhang, W.; Zheng, X.; Zhang, L.; Yao, T., Coordination-Engineered Cu–N_x Single-Site Catalyst for Enhancing Oxygen Reduction Reaction. *ACS. Appl. Energy. Mater.* **2019,** *2*, 6497-6504.
23. Jiang, Q.; Xiong, P.; Liu, J.; Xie, Z.; Wang, Q.; Yang, X. Q.; Hu, E.; Cao, Y.; Sun, J.; Xu, Y.; Chen, L., A Redox-Active 2D Metal–Organic Framework for Efficient Lithium Storage with Extraordinary High Capacity. *Angew. Chem. Int. Ed.* **2020,** *59*, 5273-5277.
24. Domınguez, C. M.; Quintanilla, A.; P.Ocn; Casas, J. A.; Rodriguez, J. J., The Use of Cyclic Voltammetry to Assess the Activity of Carbon Materials for Hydrogen Peroxide Decomposition. *Carbon.* **2013,** *60*, 76-83.

25. Kortlever, R.; Tan, K. H.; Kwon, Y.; Koper, M. T. M., Electrochemical Carbon Dioxide and Bicarbonate Reduction on Copper in Weakly Alkaline Media. *J. Solid. State. Electrochem.* **2013,** *17*, 1843-1849.
26. Kortlever, R.; Balemans, C.; Kwon, Y.; Koper, M. T. M., Electrochemical CO_2 Reduction to Formic Acid on a Pd-Based Formic Acid Oxidation Catalyst. *Catal. Today* **2015,** *244*, 58-62.
27. Chen, X.; Dong, J.; Chi, K.; Wang, L.; Xiao, F.; Wang, S.; Zhao, Y.; Liu, Y., Electrically Conductive Metal-Organic Framework Thin Film-Based On-Chip Micro-Biosensor: A Platform to Unravel Surface Morphology-Dependent Biosensing. *Adv. Funct. Mater.* **2021,** *31*, 2102855.
28. Ma, C. B.; Xu, Y.; Wu, L.; Wang, Q.; Zheng, J. J.; Ren, G.; Wang, X.; Gao, X.; Zhou, M.; Wang, M.; Wei, H., Guided Synthesis of a Mo/Zn Dual Single-Atom Nanozyme with Synergistic Effect and Peroxidase-like Activity. *Angew. Chem. Int. Ed.* **2022,** *61*, e202116170.
29. Hong, C.; Zhang, X.; Wu, C.; Chen, Q.; Yang, H.; Yang, D.; Huang, Z.; Cai, R.; Tan, W., On-Site Colorimetric Detection of Cholesterol Based on Polypyrrole Nanoparticles. *ACS. Appl. Mater. Interfaces.* **2020,** *12*, 54426-54432.
30. Wu, Q.; He, L.; Jiang, Z. W.; Li, Y.; Cao, Z. M.; Huang, C. Z.; Li, Y. F., CuO Nanoparticles Derived From Metal-Organic Gel with Excellent Electrocatalytic and Peroxidase-Mimicking Activities for Glucose and Cholesterol Detection. *Biosens. Bioelectron.* **2019,** *145*, 111704.
31. Saxena, U.; Chakraborty, M.; Goswami, P., Covalent Immobilization of Cholesterol Oxidase on Self-Assembled Gold Nanoparticles for Highly Sensitive Amperometric Detection of Cholesterol in Real Samples. *Biosens. Bioelectron.* **2011,** *26*, 3037-2043.
32. Tong, Y.; Li, H.; Guan, H.; Zhao, J.; Majeed, S.; Anjum, S.; Liang, F.; Xu, G., Electrochemical Cholesterol Sensor Based on Carbon Nanotube@Molecularly Imprinted Polymer Modified Ceramic Carbon Electrode. *Biosens. Bioelectron.* **2013,** *47*, 553-558.
33. Kozitsina, A. N.; Okhokhonin, A. V.; Matern, A. I., Amperometric Detection of Cholesterol Using Cobalt (II) Chloride as an Electrocatalyst in Aprotic Media. *J. Electroanal. Chem.* **2016,** *772*, 89-95.
34. Gautam, V.; Singh, K. P.; Yadav, V. L., Polyaniline/MWCNTs/Starch Modified Carbon Paste Electrode for Non-Enzymatic Detection of Cholesterol: Application to Real Sample (Cow Milk). *Anal. Bioanal. Chem.* **2018,** *410*, 2173-2181.
35. Ji, R.; Wang, L. L.; Wang, G. F.; Zhang, X. J., Synthesize Thickness Copper(I) Sulfide Nanoplates on Copper Rod and It's Application as Nonenzymatic Cholesterol Sensor. *Electrochim. Acta.* **2014,** *130*, 239-244.
36. Rengaraj, A.; Haldorai, Y.; Kwak, C. H.; Ahn, S.; Jeon, K. J.; Park, S. H.; Han, Y. K.; Huh, Y. S., Electrodeposition of Flower-Like Nickel Oxide on CVD-Grown Graphene to Develop an Electrochemical Non-Enzymatic Biosensor. *J. Mater. Chem. B.* **2015,** *3*, 6301-6309.

37. Khaliq, N.; Rasheed, M. A.; Cha, G.; Khan, M.; Karim, S.; Schmuki, P.; Ali, G., Development of Non-Enzymatic Cholesterol Bio-Sensor Based on TiO_2 Nanotubes Decorated with Cu_2O Nanoparticles. *Sens. Actuators. B. Chem.* **2020,** *302*, 127200.
38. Yang, L.; Zhao, H.; Li, Y.; Ran, X.; Deng, G.; Zhang, Y.; Ye, H.; Zhao, G.; Li, C. P., Indicator Displacement Assay for Cholesterol Electrochemical Sensing Using a Calix[6]arene Functionalized Graphene-Modified Electrode. *Analyst.* **2016,** *141*, 270-278.
39. Akshaya, K. B.; Varghese, A.; Nidhin, M.; George, L., Amorphous Ru-Pi Nanoclusters Coated on Polypyrrole Modified Carbon Fiber Paper for Non-Enzymatic Electrochemical Determination of Cholesterol. *J. Electrochem. Soc.* **2019,** *166*, B1016-B1027.
40. Gholivand, M. B.; Khodadadian, M., Amperometric Cholesterol Biosensor Based on the Direct Electrochemistry of Cholesterol Oxidase and Catalase on a Graphene/ionic Liquid-Modified Glassy Carbon Electrode. *Biosens. Bioelectron.* **2014,** *53*, 472-478.
41. Giri, A. K.; Charan, C.; Saha, A.; Shahi, V. K.; Panda, A. B., An Amperometric Cholesterol Biosensor With Excellent Sensitivity and Limit of Detection Based on an Enzyme-Immobilized Microtubular ZnO@ZnS Heterostructure. *J. Mater. Chem. A.* **2014,** *2*, 16997-17004.
42. Li, L.; Wang, Y.; Pan, L.; Shi, Y.; Cheng, W.; Shi, Y.; Yu, G., A Nanostructured Conductive Hydrogels-Based Biosensor Platform for Human Metabolite Detection. *Nano Lett* **2015,** *15*, 1146-1151.
43. Tripathy, N.; Ahmad, R.; Kim, E. Y.; Khang, G.; Hahn, Y. B., Cholesterol Biosensing Based on Highly Immobilized ChOx on ZnO Hollow Nanospheres. *Rsc. Adv.* **2014,** *4*, 46049-46053.
44. Dervisevic, M.; Çevik, E.; Şenel, M.; Nergiz, C.; Abasiyanik, M. F., Amperometric Cholesterol Biosensor Based on Reconstituted Cholesterol Oxidase on Boronic Acid Functional Conducting Polymers. *J. Electroanal. Chem.* **2016,** *776*, 18-24.
45. Phetsang, S.; Jakmunee, J.; Mungkornasawakul, P.; Laocharoensuk, R.; Ounnunkad, K., Sensitive Amperometric Biosensors for Detection of Glucose and Cholesterol Using a Platinum/Reduced Graphene Oxide/Poly(3-aminobenzoic acid) Film-Modified Screen-Printed Carbon Electrode. *Bioelectrochemistry.* **2019,** *127*, 125-135.
46. Zhou, X. B.; Wang, M. J.; Wang, M. K.; Su, X. G., Nanozyme-Based Detection of Alkaline Phosphatase. *Acs. Appl. Nano. Mater.* **2021,** *4*, 7888-7896.
47. Xie, X.; Wang, Y.; Zhou, X.; Chen, J.; Wang, M.; Su, X., Fe-N-C Single-Atom Nanozymes with Peroxidase-Like Activity for the Detection of Alkaline Phosphatase. *Analyst.* **2021,** *146*, 896-903.
48. Liu, W. D.; Chu, L.; Zhang, C. H.; Ni, P. J.; Jiang, Y. Y.; Wang, B.; Lu, Y. Z.; Chen, C. X., Hemin-Assisted Synthesis of Peroxidase-Like Fe-N-C Nanozymes for Detection of Ascorbic Acid-Generating Bio-Enzymes. *Chem. Eng. J.* **2021,** *415*, 128876.

49. Jiang, X. Q.; Wang, X. Y.; Lin, A. Q.; Wei, H., *In Situ* Exsolution of Noble-Metal Nanoparticles on Perovskites as Enhanced Peroxidase Mimics for Bioanalysis. *Anal. Chem.* **2021,** *93*, 5954-5962.
50. Wang, A. L.; Teng, J. X.; Yang, C. G.; Xu, Z. R., Rapid and Facile Electrospray Preparation of $CsPbBr_3$@PMMA Fluorescent Microspheres for Fluorescent Detection of ALP in Biological Samples. *Colloids. Surf.* **2022,** *634*, 127909.
51. Wang, Y. T.; Zhang, D. G.; Zhang, H.; Shang, L. R.; Zhao, Y. J., Responsive Photonic Alginate Hydrogel Particles for the Quantitative Detection of Alkaline Phosphatase. *Npg. Asia. Mater.* **2022,** *14*, 54.
52. Zhang, Q. T.; Yu, Y. Y.; Yun, X. J.; Luo, B.; Jiang, H. R.; Chen, C. Z.; Wang, S. F.; Min, D. Y., Multicolor Colorimetric Sensor for Detection of Omethoate Based on the Inhibition of the Enzyme-Induced Metallization of Gold Nanorods. *Acs. Appl. Nano. Mater.* **2020,** *3*, 5212-5219.
53. Wang, J. W.; Ni, P. J.; Chen, C. X.; Jiang, Y. Y.; Zhang, C. H.; Wang, B.; Cao, B. Q.; Lu, Y. Z., Colorimetric Determination of the Activity of Alkaline Phosphatase by Exploiting the Oxidase-Like Activity of Palladium Cube@CeO2 Core-Shell Nanoparticles. *Microchim. Acta.* **2020,** *187*, 115.
54. Li, X.; Wang, X.; Guo, W.; Wang, Y.; Hua, Q.; Tang, F.; Luan, F.; Tian, C.; Zhuang, X.; Zhao, L., Selective Detection of Alkaline Phosphatase Activity in Environmental Water Samples by Copper Nanoclusters Doped Lanthanide Coordination Polymer Nanocomposites as the Ratiometric Fluorescent Probe. *Biosensors (Basel)* **2022,** *12*, 372.
55. Su, S. X.; Hu, J.; Wang, Y. D.; Yi, J. H.; Xianyu, Y. L., Plasmonic Sensing of Alkaline Phosphatase and Its Inhibitors through Ag(I)-Mediated Assembly of Gold Nanoparticles. *Biosens. Bioelectron.* **2022,** *11*, 100208.
56. Ma, F.; Zhao, N. N.; Liu, M.; Xu, Q.; Zhang, C. Y., Single-Molecule Biosensing of Alkaline Phosphatase in Cells and Serum Based on Dephosphorylation-Triggered Catalytic Assembly and Disassembly of the Fluorescent DNA Chain. *Anal. Chem.* **2022,** *94*, 6004-6010.
57. Hayat, A.; Bulbul, G.; Andreescu, S., Probing Phosphatase Activity Using Redox Active Nanoparticles: A Novel Colorimetric Approach for the Detection of Enzyme Activity. *Biosens. Bioelectron.* **2014,** *56*, 334-339.
58. Liu, J. J.; Tang, D. S.; Chen, Z. T.; Yan, X. M.; Zhong, Z.; Kang, L. T.; Yao, J. N.; Chemical Redox Modulated Fluorescence of Nitrogen-Doped Graphene Quantum Dots for Probing the Activity of Alkaline Phosphatase. *Biosens. Bioelectron.* **2017,** *94*, 271-277.
59. Dhanshri, S.; Sahoo, S. K., Vitamin B6 Cofactor-Directed Fluorescent “Turn-On” Detection of Alkaline-Phosphatase Activity Using Bovine Serum Albumin-Functionalized Mn–ZnS Quantum Dots. *Sensors & Diagnostics* **2022,** *1*, 579-585.

60. Li, S.; Dong, Q.; Yu, Y.; Lin, B. X.; Zhang, L.; Guo, M. L.; Cao, Y. J.; Wang, Y., Redox-Responsive Breakup of a Nucleic Acids@CoOOH Nanocomplex Triggering Cascade Recycling Amplification for Sensitive Sensing of Alkaline Phosphatase. *Anal. Chem.* **2022,** *94*, 6711-6718.
61. Wang, M. Q.; Zhao, Z. H.; Gong, W. J.; Zhang, M.; Lu, N., Modulating the Biomimetic and Fluorescence Quenching Activities of Metal-Organic Framework/Platinum Nanoparticle Composites and Their Applications in Molecular Biosensing. *ACS Appl. Mater. Interfaces.* **2022,** *14*, 21677-21686.
62. Dewangan, L.; Korram, J.; Karbhal, I.; Nagwanshi, R.; Ghosh, K. K.; Pervez, S.; Satnami, M. L., Alkaline Phosphatase Immobilized CdTe/ZnS Quantum Dots for Dual-Purpose Fluorescent and Electrochemical Detection of Methyl Paraoxon. *Ind. Eng. Chem. Res.* **2022,** *61*, 3636-3646.
63. Meng, F. H. Shi, W.; Sun, Y. N.; Zhu, X.; Wu, G. S.; Ruan, C. Q.; Liu, X.; Ge, D. T., Nonenzymatic Biosensor Based on Cu_xO Nanoparticles Deposited on Polypyrrole Nanowires for Improving Detection Range. *Biosens. Bioelectron.* **2013,** *42*, 141-147.
64. Ding, L. J.; Zhao, M. G.; Fan, S. S.; Ma, Y.; Liang, J. J.; Wang, X. T.; Song, Y. W.; Chen, S., Preparing Co_3O_4 Urchin-Like Hollow Microspheres Self-Supporting Architecture for Improved Glucose Biosensing Performance. *Sens. Actuators. B. Chem.* **2016,** *235*, 162-169.
65. Umar, A.; Rahman, M. M.; AlHajry, A.; Hahn, Y. B., Enzymatic Glucose Biosensor Based on Flower-Shaped Copper Oxide Nanostructures Composed of Thin Nanosheets. *Electrochem. Commun.* **2009,** *11*, 278-281.
